# “Immunoinformatic Identification of T-Cell and B-Cell Epitopes From *Giardia lamblia* Immunogenic Proteins as Candidates to Develop Peptide-Based Vaccines Against Giardiasis”

**DOI:** 10.3389/fcimb.2021.769446

**Published:** 2021-10-27

**Authors:** Thania Garzon, David Ortega-Tirado, Gloria Lopez-Romero, Efrain Alday, Ramón Enrique Robles-Zepeda, Adriana Garibay-Escobar, Carlos Velazquez

**Affiliations:** Department of Chemistry-Biology, University of Sonora, Hermosillo, Mexico

**Keywords:** immunogenic, epitope, protection, vaccine, immunoinformatic

## Abstract

Giardiasis is one of the most common gastrointestinal infections worldwide, mainly in developing countries. The etiological agent is the *Giardia lamblia* parasite. Giardiasis mainly affects children and immunocompromised people, causing symptoms such as diarrhea, dehydration, abdominal cramps, nausea, and malnutrition. In order to develop an effective vaccine against giardiasis, it is necessary to understand the host-*Giardia* interactions, the immunological mechanisms involved in protection against infection, and to characterize the parasite antigens that activate the host immune system. In this study, we identify and characterize potential T-cell and B-cell epitopes of *Giardia* immunogenic proteins by immunoinformatic approaches, and we discuss the potential role of those epitopes to stimulate the host´s immune system. We selected the main immunogenic and protective proteins of *Giardia* experimentally investigated. We predicted T-cell and B-cell epitopes using immunoinformatic tools (NetMHCII and BCPREDS). Variable surface proteins (VSPs), structural (giardins), metabolic, and cyst wall proteins were identified as the more relevant immunogens of *G. lamblia*. We described the protein sequences with the highest affinity to bind MHC class II molecules from mouse (I-A^k^ and I-A^d^) and human (DRB1*03:01 and DRB1*13:01) alleles, as well as we selected promiscuous epitopes, which bind to the most common range of MHC class II molecules in human population. In addition, we identified the presence of conserved epitopes within the main protein families (giardins, VSP, CWP) of *Giardia*. To our knowledge, this is the first *in silico* study that analyze immunogenic proteins of *G. lamblia* by combining bioinformatics strategies to identify potential T-cell and B-cell epitopes, which can be potential candidates in the development of peptide-based vaccines. The bioinformatics analysis demonstrated in this study provides a deeper understanding of the *Giardia* immunogens that bind to critical molecules of the host immune system, such as MHC class II and antibodies, as well as strategies to rational design of peptide-based vaccine against giardiasis.

## Introduction

Giardiasis is a highly prevalent foodborne gastrointestinal parasitic infection in developing countries, mainly affecting children and immunocompromised individuals. The clinical manifestations of giardiasis vary from asymptomatic to acute or chronic episodes characterized by severe diarrhea, accompanied with abdominal pain and intestinal lesions that lead to nutrient malabsorption syndrome and weight loss (Eckmann, 2003; Cedillo-Rivera et al., 2009; Ankarklev et al., 2010; Lujan and Svard, 2011; Lopez-Romero et al., 2015). *Giardia lamblia* is the etiological agent of giardiasis, a binucleated and flagellated protozoan that can infect humans and other mammals. *G. lamblia* has a simple life cycle, consisting of two different developmental stages defined by specific structural and biochemical features, wherein the cyst is the infective form, whereas the trophozoite is the proliferative form that colonizes the upper tract of small intestine (Lujan, 2006; Cedillo-Rivera et al., 2009; Ankarklev et al., 2010; Lopez-Romero et al., 2015).

The establishment of endoparasitic infections rely on the intricate molecular interaction between each specific stage of the life cycle of parasites and the immune responses of their hosts (Tedla et al., 2019; Smith et al., 2021). Generally, the integration of innate and adaptive immune responses defines the fate of parasitic infections, therefore immunocompetence, immunopolymorphism and immunological memory of the host are important for the resolution of parasitic infections (Lima and Lodoen, 2019; Mukherjee et al., 2019).

Several studies have reported the central role of the immune system in resolution of giardiasis by using different experimental approaches (Li et al., 2004; Ankarklev et al., 2010; Kamda et al., 2012; Dreesen et al., 2014; Grit et al., 2014; Lopez-Romero et al., 2015; Singer, 2016). The mechanism of pathogen clearance mainly depend on the processes mediated by adaptive effector cells, both B and T lymphocytes. Murine models of giardiasis have demonstrated that the establishment of humoral immunity could be implicated in resolution of infection (Singer and Nash, 2000; Eckmann, 2003; Velazquez et al., 2005). In addition, the role of mucosal and circulatory CD4+ T cells has been described as essential to collaborate with the activation of B cells and control murine giardiasis (Singer and Nash, 2000; Lujan, 2011; Singer, 2016). Interestingly, whilst CD4+ T cells are important effectors in giardiasis resolution, CD8+ T lymphocyte responses have been associated to the pathophysiological damage observed during *G. lamblia* infection, such as enterocyte ultrastructural alterations, representing a paradoxical challenge for immunotherapy against giardiasis (Scott et al., 2004; Lopez-Romero et al., 2015).

The development of effective vaccines against endoparasites is limited, partially due to the complex life-cycle of parasites and the mechanisms that have acquired to successfully overcome some immune responses, such as antigenic variation, and partially to the limitations of classical vaccine design strategies (Skwarczynski and Toth, 2016; Lima and Lodoen, 2019; Moormann et al., 2019; Autheman et al., 2021; Robleda-Castillo et al., 2021). At present, there are no approved vaccines for human use against giardiasis. However, the presence of immunogenic proteins in both, cyst and trophozoite forms of *G. lamblia* have been described by different approaches. Among the proteins of *G. lamblia* able to elicit immune responses are the variable surface proteins (VSP), heat shock proteins, lectins, cyst wall proteins (CWP) and cytoskeleton associated proteins, such as giardins and tubulins (Davids et al., 2006; Lopez-Romero et al., 2017; Quintero et al., 2017).

Nowadays, synthetic peptide-based vaccines are designed considering immunodominance, epitope structure, and adjuvants to stimulate and confer protection without the complete protein or pathogen administration (Skwarczynski and Toth, 2016; Malonis et al., 2020). Immunoinformatic analysis have been used to identify immunogenic antigens from medically important protozoa, such as *Leishmania*, *Trypanosoma*, and *Plasmodium*, which have been implemented in multi-peptide vaccines with high efficacy for the control of infection. For the malaria infection, the Mosquirix ™ vaccine is currently in Clinical Trial Phase III (Teh-Poot et al., 2015; Cecilio et al., 2017; Laurens, 2020; Vakili et al., 2020).

Immunoinformatic analysis allows the identification of potential B-cell and T-cell epitopes pursued for the design of new peptide-based vaccine candidates, by combining proteomics and bioinformatics strategies. Potential B-cell epitopes are considered according to their surface accessibility, flexibility and physicochemical characteristics to interact with complementarity-determining regions (CDRs) in the antibody molecule, whereas T-cell lineal peptide epitopes are predicted based on their high-affinity binding to the major histocompatibility complex (MHC) class I and II molecules (Teh-Poot et al., 2015; Goodswen et al., 2017; Robleda-Castillo et al., 2021).

**Figure 1:**
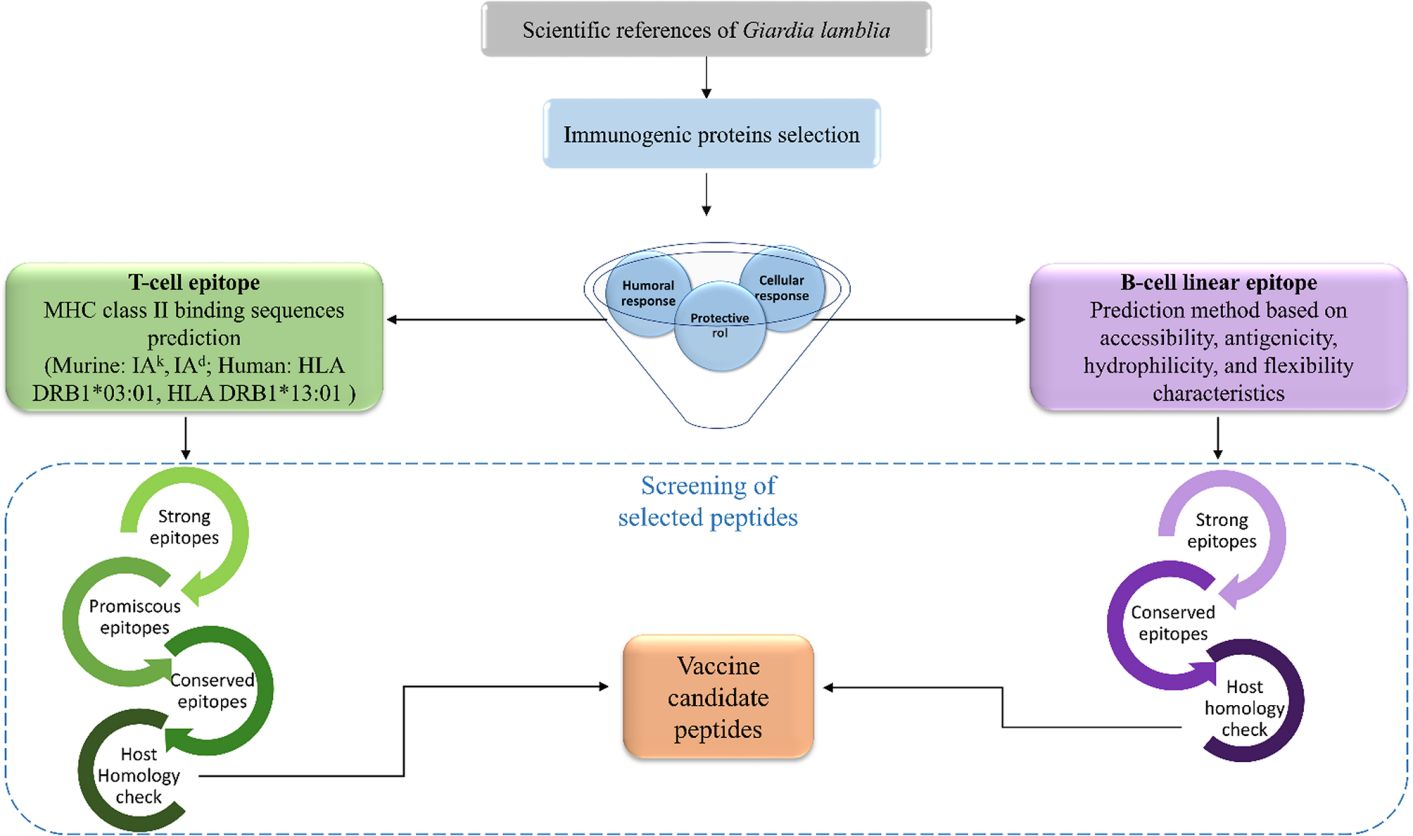
Flowchart of study design. Analysis started from the bibliographic search and selection of *G. lamblia* proteins reported as immunogenic. Prediction of T- and B-cell epitopes and screening analyses were performed to propose candidate peptides for the vaccine design, such as promiscuous and conservation epitope analysis, and host (human and mouse) homology analysis.

The aim of this study was to identify T-cell and B-cell epitopes within the immunogenic proteins of *G. lamblia* that induce a potential protective response against giardiasis, using immunoinformatic strategies (**Figure 1**). In addition, we analyzed and discussed the potential role of those epitopes to stimulate the host’s immune system, providing candidates for the development of peptide-based vaccines.

## Materials and Methods

### Search and Selection of *Giardia* Immunogenic Proteins

The identification and selection of immunogenic antigens from *Giardia* was performed on the scientific platform NCBI (PubMed: http://www.ncbi.nlm.nih.gov/pubmed/) by filtering the results to the last 30 years, using several keywords to identify the potential articles, including: *Giardia lamblia*, immunogenic proteins, protection, immune response, vaccine, variant-surface proteins (VSPs), giardins, and cyst wall proteins (CWPs). Scientific papers were selected based on their evaluations of the humoral and cellular immune response activation by *Giardia* antigens, as well as in the *in vitro* and *in vivo* protection assays. The identified *G. lamblia* immunogens were categorized according to their functionality and location in the parasite as reported in web site Uniprot (https://www.uniprot.org/) and as reported in publications. The access numbers of the selected immunogens were located in GenBank and *Giardia*DB. BLASTp analysis was performed between the assemblages of each protein.

### CD4+ T-Cell Epitope Prediction

For MHC-II-binding epitopes, 15-mer long epitopes for each protein were predicted using NetMHCIIpan 3.2 server (http://www.cbs.dtu.dk/services/NetMHCIIpan-3.2/). We selected for T-cell epitopes prediction, the murine MHC class II molecules I-A^k^ and I-A^d^. Those MHC molecules are expressed on the C3H/He and BALB/c mouse models, respectively, which are mouse strains frequently used in giardiasis studies (Belosevic et al., 1984; Venkatesan et al., 1997; Larocque et al., 2003; Lee et al., 2014; Serradell et al., 2019; Garzon et al., 2020). The HLA-DRB1*03:01 and HLA-DRB1*13:01 human MHC class II molecules were selected due to their probable association with susceptibility to infection (AL-Khaliq et al., 2020; El-Beshbishi et al., 2020). The proteins Hen Egg-white Lysozyme (HEL) and ovalbumin (Ova) were used as control antigens for the epitope prediction of MHC class II alleles (I-A^k^ and I-A^d^, respectively). The predicted peptides were classified as strong and weak binders with a threshold percentile rank (% Rank) ≤ 2% and ≤ 10%, respectively. The non-binder peptides (> 10% rank) were not considered in the study. In addition, we performed a host homology analysis. We analyzed the homology of peptides with human proteins sequence (*Homo sapiens*, taxid:9606) and mouse (*Mus musculus*, taxid:10090). The immunodominant protein sequences of *Giardia* were subjected to BLASTp against non-redundant protein sequences (nr) database (Altschul et al., 1990), and complemented with Dynamic Vaxign analysis (Xiang and He, 2009; He et al., 2010). A selection of T-cell and B-cell peptide epitopes were screened in the alignments to identify homologs. A percentage identity > 35% was set as a filter to consider homology in each epitope (Pertsemlidis and Fondon, 2001).

### Prediction of Promiscuous Peptides for MHC Class II Alleles

The analyses of epitopes with promiscuous binding to a variety of MHC class II alleles permit a greater chance of the CD4+ T cells stimulation and allow to propose ideal epitopes for a clinically effective vaccine. The identification of T-cell epitopes with promiscuous binding to MHC class II alleles was determined with the TepiTool analysis resource from the IEDB (Paul et al., 2016) (http://tools.iedb.org/tepitool/). The predictions were done by using the consensus method (Wang et al., 2008; Wang et al., 2010) which employs SMM_align, NN_align, Combinatorial library, Sturniolo methods and NetMHCIIpan (Nielsen et al., 2008; Karosiene et al., 2013). A pre-selected reference panel of 26 alleles was employed and only the peptide epitopes binding at least 50% of the alleles were selected as promiscuous (Greenbaum et al., 2011). By default, Tepitool selects the epitopes with a percentile rank ≤ 20 as promiscuous. The input sequences of epitopes were those determined as the strongest binders for murine I-A^k^, I-A^d^ alleles, HLA-DRB1*03:01 and HLA-DRB1*13:01.

### B-Cell Epitope Prediction

Linear/continuous B-cell epitopes for secreted or extracellular proteins were identified using BCPred method in BCPREDS server which is based on support vector machine (SVM) that uses string kernels (http://ailab-projects1.ist.psu.edu:8080/bcpred/predict.html) (El-Manzalawy et al., 2008). We used the following parameters for prediction, 80% specificity and a cut-off score > 0.6. Epitopes with a length of 16-mer and 18-mer were selected for the study since most B-cell epitopes are between 15 to 25 long amino acids (Potocnakova et al., 2016), also better accuracy percentages are obtained with peptide windows of 16 amino acids in length (El-Manzalawy et al., 2008).

### Epitope Conservation Analysis

To identify the percentage of conservation of the epitopes in the sequences of the proteins classified within the families, giardins, VSPs, and CWPs, the FASTA sequences of proteins were selected for a multi-alignment in T-coffee (https://www.ebi.ac.uk/Tools/msa/tcoffee/) and Boxshade webserver (https://embnet.vital-it.ch/software/BOX_form.html). The conservancies of strong T-cell epitope and B-cell epitopes previously predicted were identify by IEDB epitope conservancy analysis tool (http://tools.iedb.org/conservancy/). The conservancy of epitope sequence was assigned at > 60% for giardins and CWPs, and > 50% for VSPs. Every T-cell and B-cell epitopes that was filtered by the threshold, was subjected to cross-reactivity analysis (mouse and human).

## Results

### *Giardia* Immunogenic Proteins Selection

**Figure 2:**
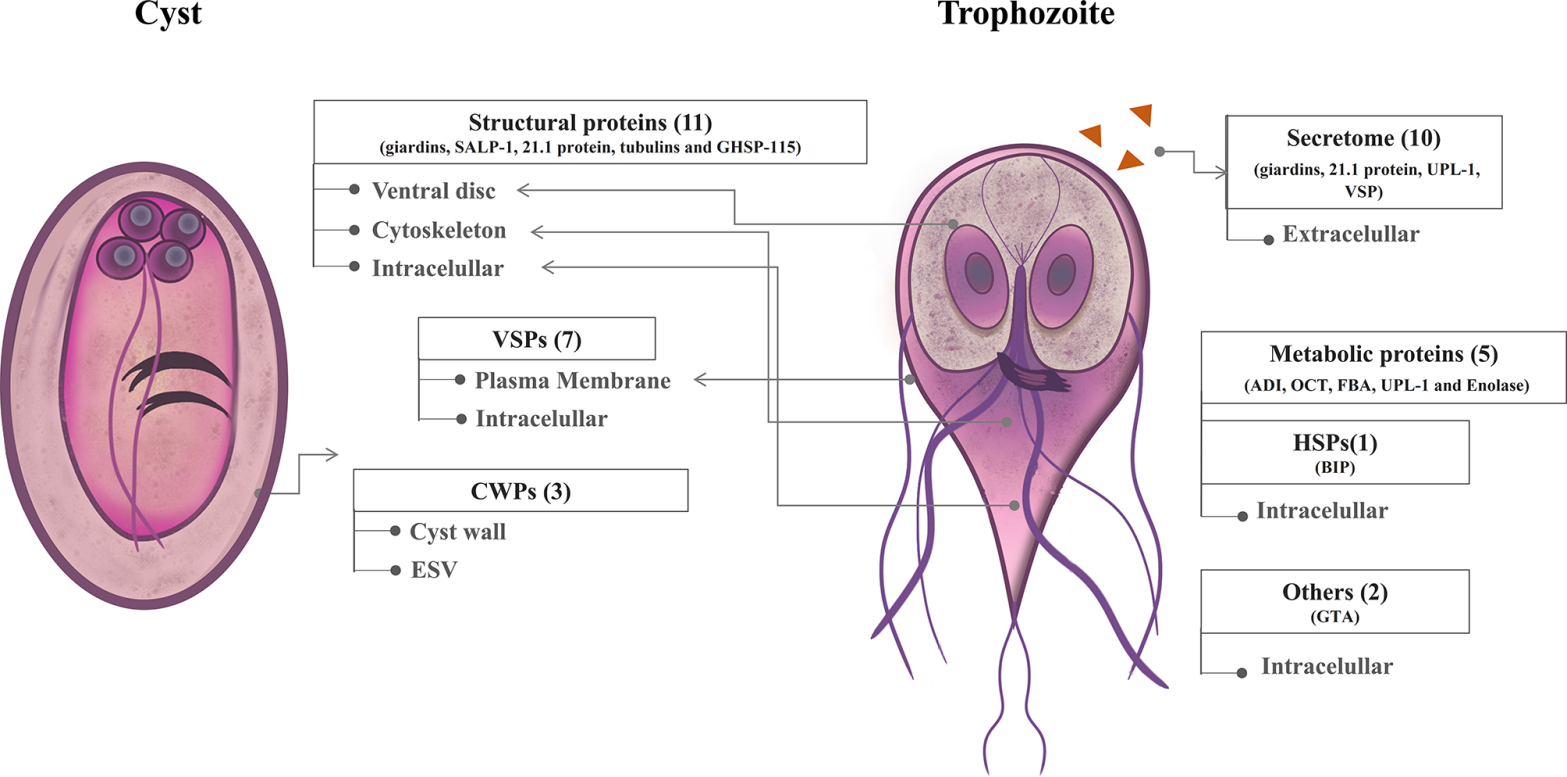
Schematic representation of cellular localization of immunogenic proteins of *G. lamblia*. A total of 29 proteins have been reported as immunogenic antigens in the cyst and trophozoite of *G. lamblia*. Immunogenic proteins were classified based on their location and function in structural proteins, metabolic proteins heat shock proteins (HSPs), variable-specific surface proteins (VSPs), and cyst wall proteins (CWPs). Proteins were located in ventral disc, plasma membrane, cytoskeleton, intracellular and secretome/extracellular of the parasite. CWPs can also be found in encystation-specific secretory vesicles (ESV).

To identify the *Giardia* immunogens, which have been described in the scientific literature, a screening search (last 30 years) of articles was performed. A total of 29 research articles were selected, wherein 29 proteins with potential high immunogenicity were reported (**Table 1**). The selected immunogens, mainly belong to WB and GS/M-83 -H7 strains, representative of *Giardia* A and B assemblages (genetic groups), respectively. The proteins presented a homology (id%) > 78% between assemblages, unlike for VSPs, due the expressed VSPs are different between the trophozoites of assemblages A and B. (Franzén et al., 2009). Proteins were classified based on their location and function (**Figure 2**). Out of the 29 immunogenic proteins identified, 3 proteins correspond to cyst wall proteins (CWP 1, CWP 2, and CWP 3), 11 proteins are structural proteins located mainly in the ventral disc and cytoskeleton, such as giardins, tubulin, SALP, 21.2 protein, and GHSP-115. In addition, 5 proteins have metabolic functions in *Giardia*, such as arginine deiminase (ADI), ornithine carbamoyl transferase (OCT), fructose-bisphosphate aldolase (FBA), uridine phosphorylase (UPL), and enolase. Among the intracellular proteins, we also found the *Giardia* Trophozoite Antigens (GTA-1 and GTA-2) and the binding immunoglobulin protein (BIP). Other immunogens in the study correspond to 7 variants-specific surface proteins (VSPs). Most of the scientific papers (more than 90%) selected during the screening search performed in the present study were focused on evaluating the immunogenicity of *Giardia* proteins by analyzing the antibody-mediated immune response. Only a few have evaluated its ability to activate cellular immune responses. The immunological assays reported in those papers have been performed using human samples, and animal models susceptible to giardia infection (mice, gerbils, kittens, and puppies). Some of those articles have reported the protective capacity of certain immunogens, such as α-1 giardin, α-11 giardin, 21.2 protein, UPL-1, VSP9B10, VSP1267, VSPH7 and CWP 2 (Larocque et al., 2003; Palm et al., 2003; Serradell et al., 2018; Davids et al., 2019; Serradell et al., 2019).

**Table 1:** Immunogenic proteins of *Giardia lamblia*.

No.	Protein	Assemblages	Id %	Location	Length(amino acids)	References
**Structural proteins**							
**1**	α-1 giardin*●	A	99%	Ventral disc	295	(Palm etal., 2003; Téllez etal., 2005; Davids etal., 2006; Feliziani etal., 2011; Jenikova etal., 2011; Feng etal., 2016; Radunovic etal., 2017; Davids etal., 2019)
		B				
**2**	α-2 giardin*	A	81%	Ventral disc	296	(Palm etal., 2003; Davids etal., 2006)
		B		Ventral disc	295	
**3**	α-7.1 giardin	A		Ventral disc	388	
**4**	α-7.3 giardin	A		Ventral disc	295	(Palm etal., 2003; Téllez etal., 2005)
**5**	α-11 giardin*●	A	91%	Ventral disc	307	(Palm etal., 2003; Davids etal., 2006; Davids etal., 2019)
		B		Ventral disc	307	
**6**	β-giardin*	A	100%	Cytoskeleton	272	(Palm etal., 2003; Téllez etal., 2005; Davids etal., 2006; Feliziani etal., 2011; Davids etal., 2019)
		B		Cytoskeleton	272	(Palm etal., 2003; Davids etal., 2006; Davids etal., 2019)
**7**	SALP-1	A	99%	Ventral disc	255	(Palm etal., 2003)
		B		Ventral disc	255	
**8**	21.1 protein*●	A	95%	Ventral disc	786	(Davids etal., 2019)
		B		Ventral disc	786	
**9**	α-Tubulin	A	100%	Cytoskeleton	754	(Palm etal., 2003; Davids etal., 2006)
		B		Cytoskeleton	754	(Palm etal., 2003)
**10**	β-Tubulin	A		Cytoskeleton	447	
**11**	GHSP-115	A		Intracelullar	1039	(Bae etal., 2009)
**Metabolic proteins**							
**12**	ADI*	A	89%	Intracelullar	580	(Palm etal., 2003; Téllez etal., 2005; Davids etal., 2006)
		B		Intracelullar	580	
**13**	OCT*	A	97%	Intracelullar	327	
		B		Intracelullar	327	
**14**	FBA*	A	97%	Intracelullar	323	
		B		Intracelullar	323	
**15**	UPL-1●	A	95%	Intracelullar	310	(Palm etal., 2003; Davids etal., 2006; Davids etal., 2019)
		B*		Intracelullar	310	
**16**	Enolase*	A	95%	Intracelullar	445	(Palm etal., 2003; Téllez etal., 2005; Davids etal., 2006; Jenikova etal., 2011)
		B		Intracelullar	445	
**Variable-specific surface proteins**							
**17**	VSP9B10*●	A		Membrane/Intracelullar	739	(Palm etal., 2003; Rivero etal., 2010; Cabrera-Licona etal., 2017; Serradell etal., 2018; Serradell etal., 2019)
**18**	VSP1267●	A		Membrane/Intracelullar	596	(Palm etal., 2003; Rivero etal., 2010; Serradell etal., 2018; Serradell etal., 2019)
**19**	VSP AS8	A		Membrane/Intracelullar	616	(Hjøllo etal., 2018)
**20**	TSA 417	A		Membrane	713	(Reiner & Gillin, 1991; Palm etal., 2003; Rivero etal., 2010)
**21**	VSPH7●	B		Membrane	557	(Stäger etal., 1997; Stäger etal., 1998; Bienz etal., 2001; Bienz etal., 2003; Serradell etal., 2018)
**22**	VSP5	B		Membrane/Intracelullar	171	(Hjøllo etal., 2018)
**23**	VSP5G8	B		Membrane	607	(Quintero etal., 2017; Garzon etal., 2020)
**Heat Shock Proteins**							
**24**	BIP	A	99%	ER/ESV	662	(Lee etal., 2014; Lopez-Romero etal., 2017)
		B		ER/ESV	677	
**Cyst Proteins**							
**25**	CWP 1	A	88%	ESV	241	(Lujan etal., 1995; Abdul-Wahid & Faubert, 2008; Ma’ayeh etal., 2017)
		B*		ESV	241	
**26**	CWP 2●	A	88%	Cyst	362	(Lujan etal., 1995; Larocque etal., 2003; Abdul-Wahid & Faubert, 2008; Lee etal., 2009; Feng etal., 2016; Radunovic etal., 2017)
		B		Cyst	363	
**27**	CWP3	A	78%	Cyst	247	(Lujan etal., 1995)
		B		Cyst	242	
**Others**							
**28**	GTA-1	A	100%	Intracellular	181	(Palm etal., 2003)
		B		Intracellular	181	
**29**	GTA-2	A	95%	Intracellular	225	(Palm etal., 2003; Davids etal., 2006)
		B		Intracellular	225	

Id %: percentage identity between G. lamblia assemblages A and B (BLAST analysis).

Giardia immunogenic proteins present in the secretome*.

Immunogenic proteins that induce protection against giardiasis ●.

### T- Cell and B-Cell Epitopes From *Giardia* Immunogenic Proteins

**Figure 3:**
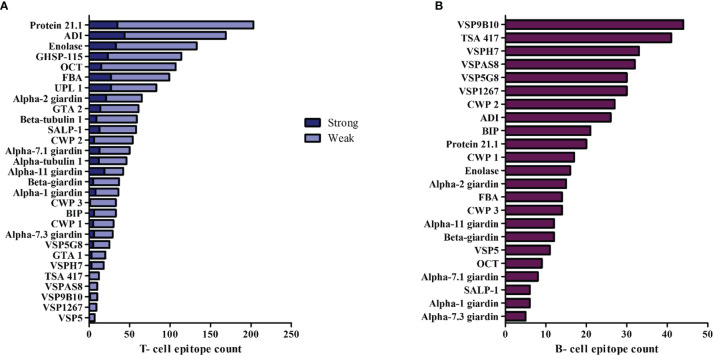
Distribution of T-cell and B- cell epitopes from *G*. *lamblia* immunogens. **(A)** T-cell epitope count for each immunogenic protein. The total count of the T- cell epitopes (strong and weak binders) was performed by the prediction in NetMHCII of MHC class II (I-A^k^, I-A^d^, DRB1*03:01, DRB1*13:01). **(B)** B-cell epitope count for each immunogenic protein. Epitope prediction of length 16 and 18 amino acids was conducted in BCPRED.

The cellular and humoral immune responses have an important role in the clearance of giardiasis. CD4+ helper T lymphocytes are involved in the activation of the effector mechanisms against *Giardia*. CD4+ cells are activated by dendritic cells, as well as by B lymphocytes through the MHC II-peptide presentation, for this reason, we initially identified T-cell epitopes from *Giardia* immunogenic proteins. T- cell epitopes that had an affinity to the murine MHC class II I-A^k^ and I-A^d^ molecules, as well as to the human MHC class II HLA-DRB1*03: 01 and HLA-DRB1*13: 01 were identified. We used the NetMHCIIpan server for T-cell epitope prediction. Out of the 29 proteins that were subjected to prediction, a total of 354 strong binder peptides and 1,298 weak binder peptides were predicted (**Figure 3**). The subsequent analyzes were focused on strong peptides. We recorded the first 5 epitopes of each protein with the highest affinity to the MHC class II molecules I-A^k^, I-A^d^, HLA-DRB1 * 03: 01 and HLA-DRB1 * 13: 01 (**Tables S1**, **S2**). Then, we selected the 20 peptide epitopes with the strongest binding affinity to each MHC class II molecule analyzed (**Tables 2**, **3**). The strong binders showed a similar percentile rank to the main immunodominant epitope (48-63) of the hen egg-white lysozyme (HEL) (Nelson et al., 1992; Velazquez et al., 2002) and to the peptide (323-339) of ovalbumin (OVA) (McFarland et al., 1999) (**Table S3**). Both peptide sequences have a high affinity binding to I-A^k^ and I-A^d^ alleles, respectively. Due to the high affinity with MHC class II molecules and the capacity to activate the cellular immune response, the binding registers of HEL and OVA peptides have been highly characterized and used as study models (McFarland et al., 1999; Bevaart et al., 2004; Dissanayake et al., 2005; Lovitch and Unanue, 2005; Landais et al., 2009; Strong and Unanue, 2011). Several sequences of giardins, UPL-1, ADI, GTA-1 and enolase showed high binding affinity for murine and human MHC II alleles (**Tables 2**, **3**). Additionally, a criterion for selection of T-cell epitopes was that they should be promiscuous. Since MHC class II alleles have different binding specificities, selection of peptides that bind to several MHC variants can allow the designing of vaccines to achieve a broad allelic coverage and protect against infection. We used the cut-off values of Tepitool to be binding to ≥ 50% of the MHC class II alleles more frequently in the world population, we found that 26 peptides sequences were highly promiscuous epitopes (**Table 4**). These data were used as screening for subsequent analyzes.

**Table 2:** Strong binder epitopes of *G. lamblia* to murine MHC class II molecules.

MHC class II I-A^k^						MHC class II I-A^d^				
Protein/Assemblage		Position	Epitope (15 mer)	Affinity (nM)	%Rank	Protein/ Assemblage	Position	Epitope (15 mer)	Affinity (nM)	%Rank
1	α-11- giardin/A,B	221	IAHYYNLAPARAVAY	3636.76	0.01	UPL 1*/B	236	AVHMSAAHIALAQRK	35.06	0.02
2	α-2- giardin/A,B	173	YISSFMAGVPPEEYK	4529.08	0.02	Enolase^ǂ^/A,B	2	EAPSTIKAIKARMII	40.24	0.03
3	GHSP-115/A,B	354	LLNEAARALPPLSPY	4967.03	0.04	UPL 1*/A	236	AVYMSAAHIALAQRK	40.3	0.03
4	ADI*/B	381	PTIDFIKASPAYISY	5149.2	0.05	α-11- giardin/B	223	HFYNLAPARAVAYAF	40.47	0.03
5	α-11- giardin/A	224	YYNLAPARAVAYAFH	5439.79	0.09	α-7.1- giardin*/A,B	14	QHLLRGATAQAAGRA	42.4	0.04
6	α-7.1- giardin*/A,B	14	QHLLRGATAQAAGRA	5456.24	0.09	α-11- giardin/A	223	HYYNLAPARAVAYAF	43.07	0.05
7	GTA 1*^ǂ^/A,B	100	LELIMSLAPNHMSAI	5487.86	0.1	UPL 1/B	234	CGAVHMSAAHIALAQ	44.58	0.05
8	α-11- giardin/B	224	FYNLAPARAVAYAFY	5635.09	0.12	α-7.1- giardin/A,B	89	SAKLKMAAAKATEIK	45.63	0.06
9	UPL 1*/B	236	AVHMSAAHIALAQRK	5650.66	0.12	Enolase/A,B	1	MEAPSTIKAIKARMI	47	0.06
10	GHSP-115/A,B	516	SDELQAARAIAEAKL	5886.75	0.17	ADI*/B	381	PTIDFIKASPAYISY	52.57	0.09
11	β -tubulin 1/A,B	272	PLTSRGSQIYRALTV	5938.82	0.2	α-7.1- giardin/A,B	91	KLKMAAAKATEIKAL	57.02	0.12
12	β -giardin/A,B	135	QIAIHNDAIAALRKE	6091.83	0.25	GTA-1*^ǂ^/A,B	100	LELIMSLAPNHMSAI	57.55	0.12
13	ADI/B	101	KYEFHPSGARITPKM	6095.59	0.25	GTA-2/B	20	VVNEIRATKVVMVSH	70.45	0.25
14	α-2- giardin/A,B	176	SFMAGVPPEEYKSIN	6227.85	0.3	BIP/A,B	152/167	EKITKAVVTVPAYFS	70.8	0.25
15	α-7.3- giardin/A,B	31	KQRAEIHAAFRAATG	6319.49	0.4	ADI/B	380	QPTIDFIKASPAYIS	71.52	0.25
16	BIP/A,B	396	DEAVAWGAAVQASIL	6320.86	0.4	GHSP-115/A,B	518	ELQAARAIAEAKLAA	72.82	0.25
17	CWP 1/A,B	91	YLSNNSLAGAIPEGL	6432.84	0.4	α-tubulin 1/A,B	326	KDVNAAIAVIKTKRT	76.23	0.3
18	UPL 1*/A	236	AVYMSAAHIALAQRK	6437.57	0.4	ADI/B	123	YKRKVLSALSTRNLV	78.29	0.4
19	ADI*/B	381	PTVDFIKADPAYISY	6470.04	0.4	Enolase/A	70	LENIRKIIAPALIGM	78.93	0.4
20	GTA-2/A,B	92	NASYHCAAAFQDSIR	6668.76	0.5	α-7.1- giardin/A,B	86	RNSSAKLKMAAAKAT	79.17	0.4

The 20 epitopes with the highest affinity to I-A^k^ and I-A^d^ MHC class II were selected.

Epitopes were organized according to % rank of affinity. Epitopes with conserved prediction with murine (*) and human (ǂ) MHC class II molecules.

**Table 3:** Strong binder epitopes of *G. lamblia* to HLA class-II molecules.

HLA class-II DRB1*03:01						HLA class-II DRB1*13:01				
Protein/Assemblage		Position	Epitope (15 mer)	Affinity (nM)	%Rank	Protein/Assemblage	Position	Epitope (15 mer)	Affinity (nM)	%Rank
1	α-2- giardin/A	4	LSQIVADMKQAIDAK	24.31	0.03	Enolase^ǂ^/A,B	2	EAPSTIKAIKARMII	9.23	0.01
2	α-2- giardin/B	4	LSQIVADIKQAIDAK	24.48	0.03	UPL 1/A,B	47	EVKFIRRAPRLFTTI	10.39	0.02
3	α- tubulin 1/A,B	112	KEIVDLVLDRVRKLA	28.47	0.06	α- tubulin 1/A,B	329	NAAIAVIKTKRTIQF	13.02	0.09
4	ADI/A,B	88	EREVLMDQAMASLKY	29.02	0.07	UPL 1/A,B	44	PGFEVKFIRRAPRLF	14.83	0.17
5	ADI/B	495	SREIIADVHKLYQKL	29.86	0.07	BIP/A,B	281/296	AKDMAVKKAISRLRR	16.24	0.25
6	GHSP-115/A,B	847	LARLRLRLDESLPAL	30.95	0.08	FBA/A	258	SRMAMTGAIRKVFVE	16.51	0.3
7	FBA/B	249	ICKINVDSDSRMAMT	31.77	0.09	FBA/B	258	SRMAMTGAIRKVFAE	16.9	0.3
8	FBA/A	249	VCKINVDSDSRMAMT	32.4	0.1	GTA 1^ǂ^/A,B	100	LELIMSLAPNHMSAI	17.37	0.4
9	CWP 1/B	60	NNYVIALDLSDMSLT	36.35	0.15	GHSP-115/A,B	660	EVIKTLRKQLVGKAT	17.63	0.4
10	α-11- giardin/B	274	WGVMRDDIISRFQSK	37.88	0.17	α-11- giardin/A	75	SARVNVIKKAMKNVN	19.64	0.5
11	FBA/A,B	251	KINVDSDSRMAMTGA	39.88	0.25	α-11- giardin/B	75	SARVNVIKKAMKGVN	19.76	0.5
12	BIP/A,B	416/431	HDVLLIDVTPLTLGI	40.31	0.25	UPL 1/B	157	LTSIVRKHVAALSYK	20.11	0.6
13	OCT/B	18	KELMYLVDRALDMKK	41.1	0.25	ADI/B	143	EPVIHLIPGVRNTAL	21.64	0.7
14	CWP 1/A	60	NNYVIALDLSDMGLT	42.32	0.25	Enolase/A	68	QALENIRKIIAPALI	22.03	0.7
15	BIP/A,B	102/117	YKVINKDGRPFVQLS	42.34	0.25	BIP/A,B	280/295	KAKDMAVKKAISRLR	22.05	0.7
16	α-11- giardin/A	274	WGVMRDDILSRFQSK	44.39	0.3	α-11- giardin/A	77	RVNVIKKAMKNVNDF	22.94	0.8
17	GHSP-115/A,B	153	KAMISHDEKTALILA	48.05	0.4	UPL 1/B	155	HDLTSIVRKHVAALS	23.41	0.9
18	α-7.3- giardin/A,B	64	LMMIVLDDEIDVRCR	48.53	0.4	α-1- giardin, α-2- giardin /A,B	244	DEKRMRRITGMMVDK	24.17	0.9
19	21.1 protein/A,B	355	NQAFKVDLNTLMSTK	49.95	0.4	Enolase/B	68	QALENIRKIITPALI	24.46	1
20	α-7.1- giardin/A,B	157	LMMIVLDDEIDVRCK	50.29	0.5	α-1- giardin, α-2- giardin/A,B	221	HFALLGMHRLAAYLI	24.69	1

The 20 epitopes with the highest affinity to HLA class-II DRB1*03:01 and DRB1*13:01 were selected.

Epitopes were organized according to % rank of affinity. Epitopes with conserved prediction with murine (*) and human (ǂ) MHC class II molecules.

**Table 4:** Promiscuous epitopes from immunogenic proteins of *G. lamblia* to MHC class II molecules.

	Epitope	Protein/Assemblage	Position	MHC II selected	Host-homol	No. Binding alleles
1	PTIDFIKASPAYISY	ADI/B	381	I-A^k^,I-A^d^	H: No M: No	20
2	QPTIDFIKASPAYIS	ADI/B	380	I-A^d^	H: No M: No	19
3	KELMYLVDRALDMKK	OCT/B	18	HLA-DRB1*03:01	H: No M: No	18
4	IAHYYNLAPARAVAY	α-11- giardin	221	I-A^k^	H: No M: No	17
5	HFYNLAPARAVAYAF	α-11- giardin/B	223	I-A^d^	H: No M: No	16
6	HYYNLAPARAVAYAF	α-11- giardin/A	223	I-A^d^	H: No M: No	16
7	KEIVDLVLDRVRKLA	α- tubulin 1	112	HLA-DRB1*03:01	H: Yes (93%) M: Yes (93%)	16
8	YYNLAPARAVAYAFH	α-11- giardin/A	224	I-A^k^	H: No M: No	16
9	LELIMSLAPNHMSAI	GTA 1	100	I-A^k^,I-A^d^ HLA-DRB1*03:01 HLA-DRB1*13:01	H: No M: No	15
10	FYNLAPARAVAYAFY	α-11- giardin/B	224	I-A^k^	H: No M: No	15
11	PGFEVKFIRRAPRLF	UPL 1	44	HLA-DRB1*13:01	H: No M: No	15
12	VVNEIRATKVVMVSH	GTA-2/B	20	I-A^d^	H: No M: No	15
13	HDVLLIDVTPLTLGI	BIP	416/431	HLA-DRB1*03:01	H: 73% M: 73%	15
14	PTVDFIKADPAYISY	ADI/B	381	I-A^k^	H: No M: No	15
15	LENIRKIIAPALIGM	Enolase/A	70	I-A^d^	H: Yes (53%) M: Yes (53%)	15
16	YKRKVLSALSTRNLV	ADI/B	123	I-A^d^	H: No M: No	15
17	AVHMSAAHIALAQRK	UPL 1/B	236	I-A^k^,I-A^d^	H: No M: No	14
18	EVKFIRRAPRLFTTI	UPL 1	47	HLA-DRB1*13:01	H: No M: No	14
19	AVYMSAAHIALAQRK	UPL 1/A	236	I-A^k^,I-A^d^	H: No M: No	14
20	LTSIVRKHVAALSYK	UPL 1/B	157	HLA-DRB1*13:01	H: No M: No	14
21	QALENIRKIIAPALI	Enolase/A	68	HLA-DRB1*13:01	H: Yes (60%) M: Yes (66%)	14
22	HFALLGMHRLAAYLI	α-1- giardin, α-2- giardin	221	HLA-DRB1*13:01	H: No M: No	14
23	EAPSTIKAIKARMII	Enolase	2	I-A^d^ HLA-DRB1*13:01	H: No M: No	13
24	NAAIAVIKTKRTIQF	α- tubulin 1	329	HLA-DRB1*13:01	H: Yes (93%) M: Yes (93%)	13
25	QALENIRKIITPALI	Enolase/B	68	HLA-DRB1*13:01	H: Yes (53%) M: Yes (60%)	13
26	LARLRLRLDESLPAL	GHSP-115	847	HLA-DRB1*03:01	H: No M: No	12

B-cell linear epitopes of *Giardia* immunogens were identified by using BCPRED tool. A total of 535 B-cell epitopes were identified (**Figure 3**). The analysis showed that the VSP membrane proteins presented a higher number of B-cell epitopes than other intracellular or cytoskeletal proteins such as giardins. The B-cell epitopes with the highest score were selected, a total of 32 epitopes were identified with a score > 0.970 (**Table 5**). Some B-cell epitopes of proteins, such as β-giardin, tubulins and VSPs obtained the maximum score issued by the BCPRED algorithm. Only in certain sequences a high homology with the proteins of human and mouse were observed, as was the case with BIP. Some predicted epitope sequences present dipeptides of proline/glycine (PG) and glutamine/proline (QP or PQ) which have been identified frequently in B-cell epitopes that induce an IgA antibody response, as well as dipeptide of alanine/serine (AG), glycine/proline (GP) and tryptophan/lysine (WK) in epitopes that activate an IgG antibody response (Gupta et al., 2013).

**Table 5:** Predicted B-cell immunodominant epitopes of *G. lamblia* proteins.

Protein		Position	Epitope(16 or 18 mer)	Score	Human homology	Mouse homology
1	β-giardin	238	DREKAERKEAEDKIVN	1.000	No	No
2	SALP-1	216	NRAIEEERAEFTEN**AG**	1.000	No	No
3	21.1 protein	24	AIPRF**AG**STNG**AG**DTG	1.000	No	No
4	α-Tubulin	438	ETLGDGEGEDMEEDDA	1.000	No	No
5	β-Tubulin	430	VDEGEEFEEEEDFGDE	1.000	No	No
6	GHSP-115	790	SV**QP**TTSTIVEEGGSD	1.000	No	No
7	FBA	274	PEKFDPRDYL**GPG**RDA	1.000	No	No
8	VSP9B10	125	AQGYFVP**PG**ADASHQS	1.000	No	No
9	VSP1267	543	TDGTSDDNSGNGGDST	1.000	No	No
10	VSP AS8	546	CAPP** AG **GS** GP **VTCYVTQQ	1.000	No	No
11	TSA 417	113	CTEAA** PG **YFAPVGAAN	1.000	No	No
12	VSPH7	371	ARAAP** PG **STPDKTNGVCT	1.000	No	No
13	VSP5	114	SCAPPTTP** PGP **VTCYV	1.000	Yes (37 %)	No
14	VSP5G8	505	CATCTTAASTCSTCAD	1.000	Yes (37 %)	Yes (37 %)
15	BIP	488	LNDIPPAPRGT** PQ **IEVTF	1.000	Yes (83 %)	Yes (83 %)
16	GTA-2	60	VPDSPPTRPSIEQLKE	1.000	No	No
17	α-11- giardin	8	PEVKAILEAKNEEEFV	0.999	No	No
18	Enolase	220	GDEGGFAPNVADPEVP	0.998	Yes (56 %)	Yes (56 %)
19	CWP 1	29	YDATDGAN** WK **TNNWLS	0.998	No	No
20	α-7.1- giardin	57	TYSPRPRTTARCDKGR	0.997	No	No
21	ADI	80	VLSEASPAEREVLMDQ	0.996	No	No
22	CWP 1	29	YDATDGAN** WK **SNNWLS	0.996	No	No
23	CWP 2	48	SYCSWTGITCDSNNNV	0.992	No	No
24	α-1- giardin	148	RVSR** PG **SPEDEAQRLD	0.991	No	No
25	CWP 3	25	FYDSTDGANWMPNNWL	0.987	No	No
26	OCT	243	MSYHITKEQKEARLKI	0.985	No	No
27	OCT	242	WMSYHITKEQKEARLK	0.978	Yes (37 %)	No
28	α-2- giardin	235	VNCACNDKGDEKRMRR	0.977	No	No
29	α-7.3- giardin	94	TDTLLTTTPEIYARVK	0.977	No	No
30	CWP 3	25	QFYDSTDGAN** WK **LNNW	0.975	No	No
31	GTA-1	162	RSIIRLPCPVSDAEVEVE	0.974	No	No
32	UPL-1	48	VKFIRRAPRLFTTITG	0.971	No	No

Bold and underline letters correspond to dipeptide regions related to activation of specific-isotype antibody response.

### Giardins, VSPs, and CWPs Have Conserved T-Cell and B-Cell Epitopes

**Figure 4:**
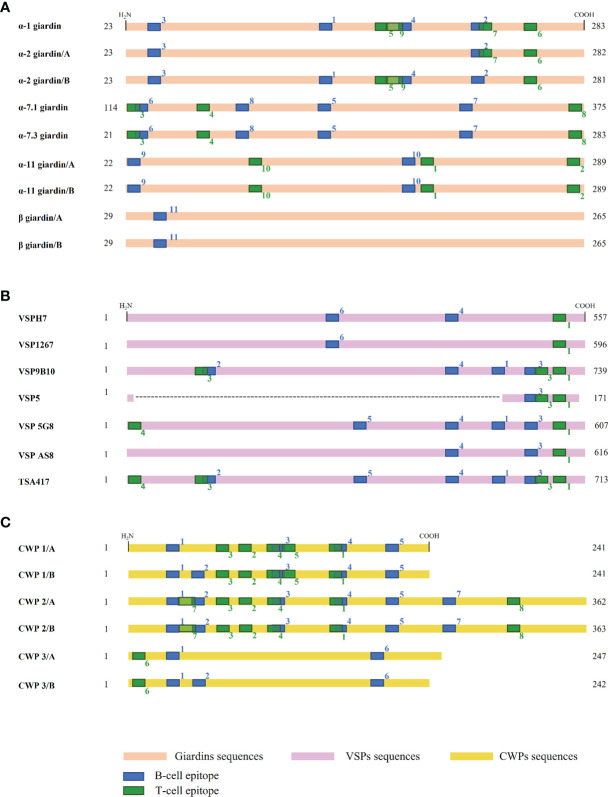
Giardins, VSPs, and CWPs conserved T-cell and B-cell epitopes. Epitope conservation analysis with a cutoff > 60% conservancy for giardins **(A)** and VSPs **(B)**, a cutoff > 50% conservancy for CWPs **(C)**. The regions shown in green, and blue are T-cell and B-cell epitopes, respectively. Conserved epitopes shown in this figure are found in **Tables 6**–**8**.

Among the immunogenic proteins identified on *Giardia*, there are three families highly characterized in the parasite, giardins, VSPs, and CWPs. It was of our interest to know whether those *Giardia* protein families conserved the predicted T-cells and B-cells epitopes. A multiple alignment of those three protein families was carried out and the epitopes that had > 60% conservation for the giardins and CWPs, and > 50% conservation for the VSPs were located. Giardins present 11 T-cell and 10 B-cell conserved epitopes (**Table 6**). The T-cell epitopes 3, 7, and 9 have amino acid residues shared with the B-cell epitopes 2, 6, and 4, respectively (**Figure 4A**). Regarding the VSPs, we identified 5 T-cell and 6 B-cell conserved epitopes (**Table 7**), which are found at the C-terminal amino acid residues. The number 1 T-cell epitope was conserved in seven proteins. In addition, the T-cell epitope 3 was the only one that overlaps with the numbers 2 and 3 of B-cell epitopes (**Figure 4B**). In the CWP family, we identified 8 and 7 T-cell and B- cell conserved epitopes, respectively (**Table 8**). Several T-cell and B-cell epitopes overlap in CWPs as T-cell epitope 1 (159-173 aa) with B-cell epitope 4 (164-181 aa) (**Figure 4C**).

**Table 6:** Epitope conservation of giardins family.

T- cell epitope							
Predicted epitope		Protein match	Epitope sequence	Position	Identity (%)	Host-homology >35%	
						Human	Mouse
1	IAHYYNLAPARAVAY	α-11 giardin/A	IAHYYNLAPARAVAY	221-235	100	No	No
		α-11 giardin/B	IAH**F**YNLAPARAVAY	221-235	100	No	No
2	WGVMRDDIISRFQSK	α-11 giardin/A	WGVMRDDI**L**SRFQSK	274-288	93.33	No	No
		α-11 giardin/B	WGVMRDDIISRFQSK	274-288	100	No	No
3	KQRAEIHAAFRAATG	α-7.1 giardin	**R**QRAEIHAAFRAAT**N**G	124-138	80	No	No
		α-7.3 giardin	KQRAEIHAAFRAATG	31-45	100	No	No
4	LMMIVLDDEIDVRCR	α-7.1 giardin	LMMIVLDDEIDVRC**K**	157-171	93.33	No	No
		α-7.3 giardin	LMMIVLDDEIDVRCR	64-78	100	No	No
5	YLIDFFGTVPSAEYR	α-1 giardin	YLIDFFGTVPSAEYR	173-187	100	No	No
		α-2 giardin/B	YLIDFFGTVPSAEYR	173-187	100	No	No
6	KYAYKTYGSMKADVE	α-1 giardin	K**H**AYK**I**YG**D**M**GT**D**I**E	263-277	60	No	No
		α-2 giardin/A	KYAYKTYGSMKADVE	263-277	100	No	No
		α-2 giardin/B	K**H**AYK**I**YG**D**M**G**AD**I**E	263-277	66.67	No	No
7	DEKRMRRITGMMVDK	α-1 giardin	DEKRMRRITGMMVDK	244-258	100	No	No
		α-2 giardin/A	DEKRMRRITGMMVDK	244-258	100	No	No
		α-2 giardin/B	DEKRMRRITGMMVDK	244-258	100	No	No
8	HYGNLAKDIRATMSK	α-7.1 giardin	HYGNLAKDIRATMSK	361-375	100	No	No
		α-7.3 giardin	HYGNLAKDIR**K**TMSK	268-282	93.33	No	No
9	RPIAEAFKAQNGKSI	α-1 giardin	RPIAEAFKAQNGKSI	187-201	100	No	No
		α-2 giardin/B	RPIAEAFKAQNGKSI	187-201	100	No	No
10	VVLIATPDERLKLAQ	α-11 giardin/A	VVLIATPDERLKLAQ	97-111	100	No	No
		α-11 giardin/B	V**I**LIATPDERLKLAQ	97-111	93.33	No	No
**B- cell epitope**							
**Predicted epitope**		**Protein match**	**Epitope sequence**	**Position**	**Identity (%)**	**Host-homology >35%**	
						**Human**	**Human**
1	RVSRPGSPEDEAQRLD	α-1 giardin	RVSR **PG** SPEDEAQRLD	143-163	100	No	No
		α-2 giardin/B	R **AS** R **PG** SPEDEAQRLD	143-163	93.75	No	No
2	INCACNDKGDEKRMRR	α-1 giardin	INCACNDKGDEKRMRR	235-250	100	No	No
		α-2 giardin/A	**V**NCACNDKGDEKRMRR	235-250	93.75	No	No
		α-2 giardin/B	INCACNDKGDEKRMRR	235-250	100	No	No
3	AKAYVASYGKELPDDIKK	α-1 giardin	AKAYV**AS**YGKELPDDIKK	39-56	100	Yes (61%)	No
		α-2 giardin/A	A**QG**Y **RDQ**YGKELPDDIKK	39-56	72.22	No	No
		α-2 giardin/B	A**QG**Y**KDQ**Y**N**KELPDDIKK	39-56	66.67	No	No
4	AEAFKAQNGKSIEQAIAT	α-1 giardin	AEAFKAQNGKSIEQAIAT	190-207	100	No	No
		α-2 giardin/B	AEAFKAQNGKSIEQAIAT	190-207	100	No	No
5	AFCRSARNNAQGDAEALK	α-7.1 giardin	AFCRSARNNAQGDAEALK	236-253	100	No	No
		α-7.3 giardin	AFCRSARNN**V**QGDAEALK	143-160	94.44	No	No
6	AEIYAAFRAANGKTASEY	α-7.1 giardin	AEIYAAFRAANGKT **AS** EY	127-144	100	No	No
		α-7.3 giardin	AEI**H**AAFRAA**T**GKT**T**SEY	34-51	83.33	No	No
7	ALCCCNATLHCPARGAAY	α-7.1 giardin	ALCCCNATLHCPARGAAY	309-326	100	No	No
		α-7.3 giardin	ALCCCNATLHCPARGAAY	216-233	100	No	No
8	TDTLLTTTPEIYARVK	α-7.1 giardin	TD**A**LLTTTPE**V**YARVK	187-202	87.50	No	No
		α-7.3 giardin	TDTLLTTTPEIYARVK	94-109	100	No	No
9	TFTSRWSAEERKELRT	α-11 giardin/A	TFTSRWSAEERKELRT	24-39	100	No	No
		α-11 giardin/B	TFTSRWSAEERKELRT	24-39	100	No	No
10	GKSVQEAIETRYADKENA	α-11 giardin/A	GKSVQEAIETRYADKENA	199-216	100	Yes (38%)	No
		α-11 giardin/B	GKSVQEAIET**K**YADKENA	199-216	100	Yes (38%)	No
11	FHDKMENEIEVRRVDD	β giardin/A	FHDKMENEIEVRRVDD	41-56	100	No	No
		β giardin/B	FHDKMENEIEVRRVDD	41-56	100	No	No

Red letters correspond to amino acids residues other than the predicted epitope.

Bold and underline letters correspond to dipeptide regions related to activation of specific-isotype antibody response.

**Table 7:** Epitope conservation of VSP family.

T- cell epitope							
Predicted epitope		Protein match	Epitope sequence	Position	Identity (%)	Host- homology >35%	
						Human	Mouse
1	LSTGAIAGISVAAFV	VSP 5G8	LS**S**GAIAGISVA**VI**V	574-588	80	No	No
		VSPH7	LS**S**GAIAGISVA**VI**V	524-538	80	No	No
		VSP9B10	LS**A**GAIAGI**A**VA**VII**	706-720	66.67	No	No
		VSP1267	LSTGAIAGISVAAFV	563-577	100	No	No
		VSP AS8	LSTGAIAGISVA**VVA**	583-597	80	No	No
		VSP5	LS**S**GAIAGISVA**VIA**	147-161	73.33	Yes (46%)	No
		TSA417	LSTGAIAGISVA**VI**V	574-588	86.67	No	No
2	SRCNTGFVPINGQCA	VSP9B10	SRCNTGFVPINGQCA	51-65	100	Yes (40%)	No
		VSP1267	**MQ**CN**Q**G**K**VPING**I**C**T**	39-53	60	No	No
3	PVLCYLVRDSASVNK	VSP 5G8	PVLCYLVRDSASVNK	557-571	100	No	No
		VSP9B10	**S**VLCYLV**QSGENT**NK	689-703	53.33	No	No
		TSA417	**S**VLCYL**IK**DS**G**S**T**NK	663-677	66.67	No	No
4	VAVILQIARAACTPG	VSP 5G8	VAVILQIARAACTPG	8-22	100	No	No
		TSA417	**AI**VILQ**L**AR**T**ACT**QE**	8-22	60	No	No
5	QAAQGYFVPPGADAS	VSP9B10	QAAQGYFVPPGADAS	123-137	100	No	No
		TSA417	**E**AA**P**GYF**A**P**V**GA**ANT**	115-129	53.33	No	No
**B- cell epitope**							
**Predicted epitope**		**Protein match**	**Epitope sequence**	**Position**	**Identity (%)**	**Host- homology >35%**	
						**Human**	**Mouse**
1	CATCTTAASTCSTCAD	VSP 5G8	CATCTTA **AS** TCSTCAD	505-520	100	Yes (37%)	Yes (37%)
		VSP9B10	CATC**AGS ASN**C**D**TC**ST**	635-650	56.25	Yes (50%)	Yes (43%)
		TSA417	C**E**TC**NG**AA**T**TC**KA**CA**T**	608-623	56.25	Yes (50%)	Yes (50%)
2	AQGYFVPPGADASHQS	VSP9B10	AQGYFV **PP** GAD **AS** HQS	125-140	100	No	No
		TSA417	A**P**GYF**A**P**V**GA**ANTE**QS	117-132	56.25	No	No
3	SCAPPTTPPGPVTCYV	VSP 5G8	SCA**A**P**SGSTGP** V**L**CY**L**	547-562	56.25	No	No
		VSP9B10	**N**CA **PP **LNNK**** G**S**V**L**CY**L**	679-694	50	No	No
		VSP AS8	SCA **PPAGSSGP** VTCYV	545-560	75	No	No
		VSP5	SCA **PP** TT **PPGP** VTCYV	114-129	100	Yes (37%)	No
		TSA417	**N**CA **PPPNNK**G**S**V**L**CY**L**	653-668	50	No	No
4	PGSTVCVTAPTGGTCT	VSP 5G8	PGSTVCVTAPTGGTCT	438-453	100	Yes (37%)	No
		VSPH7	PG**N**T**L**C**T**TA**DA**G**KCT**T	408-423	50	No	Yes (37%)
		VSP1267	**A**G**RD**V**S**V**CTA**TGG**K**CT	406-421	50	No	No
		VSP AS8	PGS**S**VC**TA**A**QNK**G**Q**CQ	464-479	50	No	No
		TSA417	PG**K**TVC**IS**AP**N**GGTC**Q**	543-558	68.75	No	No
5	KGATASDCTACPAGRA	VSP 5G8	KGAT **AS** DCTACPAGRA	329-344	100	Yes (50%)	Yes (43%)
		TSA417	**SAG**T **AS** DCT**E**CP**T**G**K**A	433-448	62.50	Yes (43%)	Yes (37%)
6	TDCPAGTYAVSGDSGS	VSPH7	**Q**DCPAGTYA**D**S**NVCKP**	272-287	56.25	Yes (62%)	Yes (50%)
		VSP1267	TDCPAGTYAVSGDSGS	303-318	100	Yes (50%)	Yes (57%)

Red letters correspond to amino acids residues other than the predicted epitope.

Bold and underline letters correspond to dipeptide regions related to activation of specific-isotype (IgG and IgA) antibody response.

**Table 8:** Epitope conservation of CWP family.

T-cell epitopes							
Predicted epitope		Protein match	Epitope sequence	Position	Identity (%)	Host- homology >35%	
						Human	Mouse
1	LKELHLDCNQLTGDV	CWP 1/A	LKELHLDCNQL**S**G**T**V	159-173	86.67	Yes (60%)	Yes (53%)
		CWP 1/B	LKELHLDCNQLTGDV	159-173	100	Yes (60%)	Yes (60%)
		CWP 2/A	LKELHLDCN**E**LTGDV	159-173	93.33	Yes (53%)	Yes (40%)
		CWP 2/B	LKELHLDCN**E**LTGDV	159-173	93.33	Yes (53%)	Yes (40%)
2	YLSNNSLAGAIPEGL	CWP 1/A	YLSNNSLAGAIPEGL	91-105	100	Yes (53%)	Yes (53%)
		CWP 1/B	YLS**S**N**T**L**T**G**D**IPEGL	91-105	73.33	Yes (46%)	Yes (40%)
		CWP 2/A	YL**N**NN**D**LAG**P**IP**TD**L	91-105	66.67	Yes (46%)	Yes (46%)
		CWP 2/B	YL**N**NN**D**LAG**P**IP**TD**L	91-105	73.33	Yes (40%)	Yes (46%)
3	DLSDMSLTGAIPENI	CWP 1/A	DLSDM**G**LTG**T**IPENI	67-81	86.67	Yes (46%)	Yes (46%)
		CWP 1/B	DLSDMSLTGAIPENI	67-81	100	No	Yes (46%)
		CWP 2/A	DLSDM**G**LTGA**L**P**AD**I	67-81	73.33	Yes	No
		CWP 2/B	DLSDM**G**LTGAIP**TD**I	67-81	80	No	No
4	LTNLQYLQINKAGLT	CWP 1/A	LTNLQYLQ**V**N**S**AGLT	108-122	86.67	No	Yes (46%)
		CWP 1/B	LTNLQYLQINKAGLT	108-122	100	Yes (40%)	Yes (40%)
		CWP 2/A	LT**SM**QYLQIN**N**AGLT	108-122	80	Yes (53%)	Yes (46%)
		CWP 2/B	LT**SM**QYLQIN**N**AGLT	108-122	80	No	Yes (46%)
5	IPECICDLTHMMFWY	CWP 1/A	IPEC**M**CDL**I**H**L**MFWY	125-139	80	No	No
		CWP 1/B	IPEC**M**CDL**V**H**L**MFWY	125-139	80	No	No
		CWP 2/A	IPECICDLTHMMFWY	125-139	100	No	No
		CWP 2/B	IPECICDLTHMMFWY	125-139	100	No	No
6	IEIGYGLADAQHDAL	CWP 3/A	**L**E**V**GYGL**V**D**M**Q**Y**DAL	8-22	66.67	No	No
		CWP 3/B	IEIGYGLADAQHDAL	9-23	100	No	No
7	WKSNNWLAADVSYCS	CWP 2/A	WKSNNWL**TP**DVSYCS	37-51	86.67	No	No
		CWP 2/B	WKSNNWLAADVSYCS	37-51	100	No	No
8	GNASRSAVARPTARA	CWP 2/A	GNASRSAVARPTARA	321-335	100	No	No
		CWP 2/B	G**S**ASRS**TTS**RPTARA	321-335	73.33	No	No
**B- cell epitope**							
**Predicted epitope**		**Proteins match**	**Epitope sequence**	**Position**	**Identity (%)**	**Host- homology >35%**	
						**Human**	**Mouse**
1	YDATDGANWKTNNWLS	CWP 1/A	YDATDGAN **WK** TNNWLS	29-44	100.	No	No
		CWP 1/B	YDATDGAN **WKS**NNWLS	29-44	93.75	No	No
		CWP 2/A	YDA**L**DGAN **WKS**NNWL**T**	29-44	81.25	No	No
		CWP 2/B	YDA**L**DGAN **WKS**NNWL**A**	29-44	81.25	No	No
		CWP 3/A	YD**S**TDGANW**MP**NNWL**Q**	26-41	75	No	No
		CWP 3/B	YD**S**TDGAN **WKL**NNWL**Q**	27-42	81.25	No	No
2	SYCSWTGITCDSNNNV	CWP 1/B	S**I**C**T**WTG**V**TCD **AS** NN**Y**	47-62	62.50	No	No
		CWP 2/A	SYCSWTGITCDSNNNV	48-63	100	No	No
		CWP 2/B	SYCSWTGITCDSNNNV	48-63	100	No	No
		CWP 3/B	**D**YC**E**WTG**VS**CD**D**NNNV	45-60	68.75	No	No
3	LQINNAGLTGDIPECI	CWP 1/A	LQI**V**N**S**GLTGDIPEC**M**	114-129	81.25	No	Yes (40%)
		CWP 1/B	LQIN**K**AGLTG**S**IPEC**M**	114-129	81.25	Yes (37%)	Yes (37%)
		CWP 2/A	LQINNAGLTGDIPECI	114-129	100	Yes (43%)	Yes (37%)
		CWP 2/B	LQINNAGLTGDIPECI	114-129	100	Yes (50%)	Yes (37%)
4	LDCNQLTGDVPVGLMTLP	CWP 1/A	LDCNQL**S**G**T**VPVGLMTLP	164-181	88.89	Yes (61%)	Yes (44%)
		CWP 1/B	LDCNQLTGDVPVGLMTLP	164-181	100	Yes (61%)	Yes (44%)
		CWP 2/A	LDCN**E**LTGDVP**AD**L**FD**LP	164-181	72.22	Yes (44%)	No
		CWP 2/B	LDCN**E**LTGDVP**AD**L**FD**LP	164-181	72.22	Yes (44%)	No
5	TTDCDYCTALPPTNCPTT	CWP 1/A	**DV**DC**EN**C**GT**L **PP** TNC**AQC**	210-227	50	Yes 38%)	Yes (39%)
		CWP 1/B	**DV**DCD**D**C**GT**L **PP** TNC **PQC**	210-227	61.11	No	No
		CWP 2/A	TTDCDYCTAL **PP** TNCPTT	211-228	100	No	No
		CWP 2/B	TTDCDYCTAL **PP** TNCP**E**T	211-228	94.44	No	No
6	ACGSNHCNNCVEKTTC	CWP 3/A	ACG**E**NHC**ST**CV**K**KTTC	205-220	75	No	No
		CWP 3/B	ACGSNHCNNCVEKTTC	206-221	100	No	No
7	CNARSASNCGKAKSNMHN	CWP 2/A	CNARS **AS** NCGKAKSNMHN	252-269	100	No	No
		CWP 2/B	CNARS **AS** NCGKAKSNMHN	252-269	100	No	No

Red letters correspond to amino acids residues other than the predicted epitope.

Bold and underline letters correspond to dipeptide regions related to activation of specific-isotype (IgG and IgA) antibody response.

## Discussion

Vaccine development has evolved over the years since Edward Jenner introduced the smallpox vaccine in 1796. Nowadays, in order to generate specific and safe vaccines with fewer side effects, extensive research is needed to design vaccines. In the initial phases, it is necessary to understand pathophysiology of infection, the pathogen-host relationship, as well as also to identify and characterize the immunodominant antigens that can generate immunity. Each research focused on those aspects supports the design of effective and safe vaccines for the population. At present, there is no vaccine for human giardiasis. Therefore, in this study, several T-cell and B-cell epitopes of *G. lamblia* immunogens were identified, which presented different immunogenic characteristics, some T-cell epitopes were promiscuous with strong binding affinity to MHC class II molecules, epitopes without homology to the hosts and conserved among protein families.

The *G. lamblia* antigens shown in **Table 1** are molecules that have been experimentally characterized as immunogens. In addition to the physicochemical properties, other characteristics contribute to the immunogenicity of a molecule. i) Foreignness of the immunogen: there must be a degree of phylogenetic difference between the candidate molecule and the host to avoid self-reactivity (Crumpton, 1974). In the present study, several amino acid regions of *G. lamblia* immunogens (tubulin, enolase, BIP, CWP and VSP) showed some degree of homology with human and mouse molecules, ubiquitous proteins in eukaryotic cells. Although, those peptides could potentially cause an autoimmune or allergenic reaction, suggesting the necessity to do additional studies to evaluate the safety of those predicted peptides. ii) Exposure to the immune system: *Giardia* is a non-invasive parasite of the intestinal mucosa, therefore extracellular proteins play an important role in stimulating the immune system (Troeger et al., 2007; Cotton et al., 2011). The antigen location is crucial for easy recognition by the immune response, which is why surface proteins have been targets for vaccine development (Serradell et al., 2016; Abdi et al., 2019; Uwase et al., 2020). *Giardia* proteins that are located in the cytoskeleton, ventral disc, membrane, and proteins of secretome (proteins with an asterisk in **Table 1**) can have greater accessibility for the immune system, activating an efficient antibody-mediated response, as well as antigen uptake and presentation by antigen-presenting cells (Kaufmann and Hess, 1999; Foged et al., 2005; Mora and Telford, 2010). iii) Chemical stability and conservation of proteins: Giardins are a large group of structural proteins that are divided into alpha, beta, and gamma, there are 21 genes for alpha-giardins that are conserved in assemblages A and B (Feliziani et al., 2011). CWP 1, CWP 2, and CWP 3 have around 60% identity in a sequence of 26 kDa, as well as conserved regions rich in leucine and cysteine (Lujan et al., 1995; Sun et al., 2003; Abdul-Wahid and Faubert, 2004). VSPs also have multiple cysteine domains, which also make them resistant to reduction by proteases (Nash et al., 1991; Papanastasiou et al., 1997). Besides, the three families of proteins mentioned above have been found expressed in the early stages of the encystation/excystation of *G. lamblia* (McCaffery et al., 1994; Hehl et al., 2000; Carranza et al., 2002; Weiland et al., 2003).

The characterization of immune responses induced by *Giardia* immunogens has mainly focused on IgA and IgG humoral response, perhaps due to the accessibility and feasibility of *in vitro* immunological assays, together with the evaluation in experimental animals. Infected mice with *G. lamblia* have demonstrated the establishment of humoral immunity around the third to fifth week post-infection, which could be implicated in the resolution of infection (Singer and Nash, 2000; Eckmann, 2003; Velazquez et al., 2005). We identified 24 immunodominant B-cell epitopes from immunogenic proteins of *Giardia* by bioinformatic analysis. Several studies have demonstrated the high immunogenicity of excretory/secretory proteins of *Giardia* (Palm et al., 2003; Hanevik et al., 2011; Jiménez et al., 2014). The metabolic proteins ADI, OCT, and enolase were recognized by serum from patients with acute giardiasis (Palm et al., 2003). In other microorganisms, the immunological role of those proteins has been evaluated. Enolase has a protective role in candidiasis (Montagnoli et al., 2004). OCT activates an antibody response in *Streptococcus suis* infection, and it is involved in reducing pathogenicity factors (Wang et al., 2020). VSPs are highly expressed on the membrane of *Giardia* trophozoite and are involved in the antigenic variation of the parasite. Although the mechanisms that induce antigenic switching are unknown, it is hypothesized that anti-VSP antibodies could stimulate the VSP switching. Several studies indicate the high effectiveness of VSPs to activate an antibody-mediated response in infected humans and animals (Stäger et al., 1998; Hjøllo et al., 2018; Serradell et al., 2018), as well as the effector mechanisms of anti-VSP antibodies against trophozoites, such as cytotoxicity, opsonization, and neutralization (Nash and Aggarwal, 1986; Stäger et al., 1997; Rivero et al., 2010).

The clearance of *Giardia* infection requires humoral and cellular immune mechanisms. In *Giardia*, there is little research focused on characterizing the cellular response, however, it is known that CD4+ T lymphocytes play an important role in infection. CD4-deficient mice treated with an anti-CD4 antibody could not clear the infection, as well as CD4+ T cells deficiency is related to chronic giardiasis (Heyworth et al., 1987; Singer and Nash, 2000). In this study, 26 epitopes are proposed to activate CD4+ cells due to their high affinity to several MHC class II molecules. First, four MHC class II alleles were chosen, the MHC class II I-A^k^ and I-A^d^ that are expressed in mice widely used as model for giardiasis, as well as the HLA-DRB1 * 03: 01 and HLA-DRB1 * 13: 01 alleles that are related to an increased risk of *G. lamblia* infection (AL-Khaliq et al., 2020; El-Beshbishi et al., 2020). MHC class II molecules are expressed in dendritic cells and B lymphocytes, which are chemoattracted by trophozoite-stimulated epithelial cells (Roxström-Lindquist et al., 2005). Dendritic cells pre-stimulated with *Giardia* antigens can confer IL-6-dependent protection, which has been related to B-lymphocyte growth and T-cell differentiation (Weaver et al., 2006; Kamda et al., 2012). Proinflammatory chemokines, including TNF-α, and B lymphocyte activating interleukins, such as IL-4 and IL-5 belong to the chemokine profile described in *Giardia* infection (Cotton et al., 2015; Serradell et al., 2018). Additionally, an increase in IL-17 producing CD4+ cells from infected patients with *Giardia* (Saghaug et al., 2015). Interleukin IL-17 has been associated with IgA production and infection control (Dann et al., 2015). Although we focused the analysis on the strong epitopes classified by the NetMHCII algorithm, we did not disregard sequences with low affinity to MHC class II for future tests, due peptides with a low binding affinity can activate effective T-cell response, as the HEL 20-35 peptide (Nelson et al., 1992; Velazquez et al., 2002).

In this study, we identified conserved epitopes among giardins, VSPs, and CWPs. T-cell and B-cell epitopes overlap in some amino acid residues. Responses between B and T cells are closely linked for the development of an effective immune response. B cells as an antigen presenting cell can recognize antigens through the BCR, as well as present T-dependent antigens through the binding of peptides to MHC class II molecules. T-helper cells recognize peptide-MHC class II complex and send activation signals to the B cell (Shimoda and Koni, 2007; Akkaya et al., 2019). Those pathways promote processes such as the isotype switch, affinity maturation and immunological memory, necessary in the development of protective immune responses. Does it remain to be evaluated whether these epitopes can be generated naturally? Are they resistant to antigen processing? if the antibodies generated are limited to the course of the infection or will it be a long-lived response?. In the analysis of multiple alignments between the proteins showed several semi-conserved epitopes in the three protein families (giardins, CWPs and VSPs), although we understand that changes in amino acids can reduce the binding affinity with MHC class II and immunoglobulins, these regions can be targets for a vaccine that protects by dampening the variations that occur in the parasite throughout its life cycle.

The proteins described in this study have been proven to be immunogenic, however, only few of them have been evaluated in protection assays. Prior to consider an immunogen as a vaccine candidate, it is crucial to demonstrate its protective capacity by using experimental models. The proteins α-1 giardin, α-11 giardin, 21.2 protein, UPL-1, VSP9B10, VSP1267, VSPH7 and CWP 2 have shown to induce a protective immune response against infection by *G. lamblia* (**Table 1**) when administered orally or intraperitoneally. Mice and Mongolian gerbils were commonly used as animal models in protection assays, although other *Giardia*-susceptible animals, such as cats and dogs have also been used (Serradell et al., 2016). Currently, there is no human or dog effective vaccine against *G. lamblia*. In 1999, Fort Dodge Animal Health developed a vaccine based on killed disrupted trophozoites (*Giardia*Vax), which attenuated giardiasis symptoms, produced antibodies, and reduced the shedding cysts to 30% and 5% in vaccinated kitten and puppies respectively (Olson et al., 2000). *Giardia*Vax was also tested on *Meriones unguiculatus*, showing protection in 33% of the mice at the third day post-infection, the rest of the vaccinated group cleared the infection at seventh day (Jiménez-Cardoso et al., 2002). However, other studies differ in the vaccine efficacy. It was reported that the *Giardia* parasite persisted by week 28 in vaccinated cats with three doses of *Giardia*vax (Stein et al., 2003), as well as in dogs, no differences were found in the elimination of cysts between the control and vaccinated group (Anderson et al., 2004). First-generation vaccines, such as the whole pathogen vaccine are characterized by generating no or low cell-mediated response and can also generate adverse effects such as hypersensitivity (Jiskoot et al., 2019). In recent years, protection strategies for clinically relevant pathogens have been focused on the peptide- and epitope-based vaccines (**Table S4**). Initially, *in silico* analysis facilitate the identification of T- and B-cell epitopes, and which can significantly reduce time and cost of research. Peptide-based vaccines can generate an effective and targeted immune response if the proper adjuvants and delivery system are considered. In gastrointestinal infections such as giardiasis several mucosal adjuvants can be used, such as choleric toxin, which increase the permeability of the intestinal epithelium promoting the antigen-uptake by immune cells (Rhee et al., 2012).

Validation strategies for the effectiveness of a peptide-based vaccine can be completed with additional *in silico* and experimental assays. In several viral pathogens, IFN-γ response activation is evaluated, due to the importance of this cytokine in effector mechanisms. Additionally, 3-D modeling and molecular docking are performed for the multi-epitopes vaccine constructs. All subunit- and epitope-based vaccines shown in **Table S4** have high protection efficacy in their respective diseases, as well as induced specific humoral and cellular responses. The advances in vaccines design of parasites show methodological strategies for the antigens characterization that can be implemented in *Giardia* studies. Likewise*, Giardia* shares some characteristics with other protozoa. *Giardia* presents antigenic variation, characteristic of the differential expression of variable surface proteins (VSP). *Plasmodium* and *Trypanosoma* are other parasites that express variable surface antigens (Borst and Ulbert, 2001; Kyes et al., 2007). Heat shock proteins are highly conserved molecules, in *Leishmania* which have been described as immunomodulatory proteins as well as have been used as components of vaccines (Lopez-Romero et al., 2017). Although there is little research on the immunological characterization of *Giardia* HSPs, studies have described the BIP protein as an immunogenic protein (Lee et al., 2014). We believe that more studies are needed to analyze the similarities among immunogenic antigens of *Giardia* and other pathogens, as well as the immune responses that may activate.

Our study is restricted by limiting immunoinformatic analyses to *G. lamblia* immunogenic proteins. At present, proteins are the molecules most characterized at the immunological level, however, different types of antigens may contribute to elicit immune responses during *G. lamblia* infection. Trophozoites of *G. lamblia* are able to activate innate immune responses, such as the complement system through the lectin pathway, after the recognition of surface N-acetylglucosamine (GlcNAc) by mannose-binding lectin (MBL). Interestingly, specific surface glycoconjugate antigens, glycosylphosphatidylinositol (GPI) and lipophosphoglycan (LPG) have been described as important inducers of immune responses during parasitic infection with *Trypanosoma spp, Leishmania spp, Plasmodium falciparum, Cryptosporidium* y *Entamoeba histolytica* (Ropert and Gazzinelli, 2000; Priest et al., 2003; Wong-Baeza et al., 2010). Based on this information, it is necessary to address future analyses at the molecular basis under the immune responses elicited by GlcNAc and other glycoconjugate antigens present in trophozoites and cysts of *G. lamblia*, in addition, the immunogenic role of post-translational modifications, such as glycosylation in VSPs, should be fully analyzed.

The present study describes a global approach to the identification of immunodominant and protective antigens of *Giardia*, being the first study to determine potential T-cell and B-cell immunogenic epitopes predicted by immunoinformatic tools as candidates for a vaccine against *Giardia* infection. For effective vaccine development against *Giardia*, it is necessary to consider several factors: i) the inclusion of conserved and variable protein sequences from the most common *Giardia* assemblages in humans (A and B); ii) the activation of the immune mechanisms of innate and adaptive response, considering the relationship between the parasite, gut mucosal immune system, microbiota, and the tolerogenic environment.: iii) the use of proper adjuvants; iv) administration routes to guarantee an immune response in mucosa. For future studies, *in vitro* and *in vivo* assays are required to verify the effectiveness and protective role of T-cell and B-cell epitopes in giardiasis. These results obtained in the present study suggest that experimental administration of a multi-epitope vaccine constructed on basis of immunoinformatic approach could provide an effective prophylactic strategy against *Giardia.*

## Data Availability Statement

The original contributions presented in the study are included in the article/**Supplementary Material**. Further inquiries can be directed to the corresponding author.

## Author Contributions

TG performed and is involved in immunoinformatic analyses, wrote the manuscript, and prepared all figures. GL-R and DO-T performed *in silico* assays and analyzed the data. EA contributed to the writing and editing of the manuscript. AG-E contributed to the writing and editing of the manuscript. RR-Z contributed to the library searches and assembling relevant literature. CV designed and supervised the project, revised the manuscript, and was responsible for the funding. All authors contributed to the article and approved the submitted version.

## Funding

National Council for Science and Technology of Mexico (CONACyT, CB2017-2018 A1-S-21831).

## Conflict of Interest

The authors declare that the research was conducted in the absence of any commercial or financial relationships that could be construed as a potential conflict of interest.

## Publisher’s Note

All claims expressed in this article are solely those of the authors and do not necessarily represent those of their affiliated organizations, or those of the publisher, the editors and the reviewers. Any product that may be evaluated in this article, or claim that may be made by its manufacturer, is not guaranteed or endorsed by the publisher.

## References

[B1] Abdi R. D. Dunlap J. R. Gillespie B. E. Ensermu D. B. Almeida R. A. Dego O. K. (2019). Comparison of Staphylococcus Aureus Surface Protein Extraction Methods and Immunogenicity. Heliyon 5 (10), e02528. doi: 10.1016/j.heliyon.2019.e02528 31687478 PMC6820086

[B2] Abdul-Wahid A. Faubert G. M. (2004). Similarity in Cyst Wall Protein (CWP) Trafficking Between Encysting Giardia Duodenalis Trophozoites and CWP-Expressing Human Embryonic Kidney-293 Cells. Biochem. Biophys. Res. Commun. 324 (3), 1069–1080. doi: 10.1016/j.bbrc.2004.09.167 15485664

[B3] Abdul-Wahid,.A. Faubert G. (2008). Characterization of the Local Immune Response to Cyst Antigens During the Acute and Elimination Phases of Primary Murine Giardiasis. Int. J. Parasitol. 38 (6), 691–703. doi: 10.1016/j.ijpara.2007.10.004 18037419

[B4] Akkaya M. Kwak K. Pierce S. K. (2019). B Cell Memory: Building Two Walls of Protection Against Pathogens. Nat. Rev. Immunol. 20, 4, 229–238. doi: 10.1038/s41577-019-0244-2 31836872 PMC7223087

[B5] AL-Khaliq I. M. A. Ghadban M. M. Karimau K. A. A. L. H. (2020). Influence of Human Leukocyte Antigen Hla-Drb1 on Susceptibility To Giardia Lamblia Infection of Iraqi Patients. Biochem. Cell. Arch. 20 (2), 5513–5516.

[B6] Altschul S. F. Gish W. Miller W. Myers E. W. Lipman D. J. (1990). Basic Local Alignment Search Tool. J. Mol. Biol. 215 (3), 403–410. doi: 10.1016/S0022-2836(05)80360-2 2231712

[B7] Anderson K. A. Brooks A. S. Morrison A. L. Reid-Smith R. J. Martin S.W. Benn D. M.. (2004). Impact of Giardia Vaccination on Asymptomatic Giardia Infections in Dogs at a Research Facility. Can. Vet. J. 45 (11), 924–930. 15600158 PMC545982

[B8] Ankarklev J. Jerlström-Hultqvist J. Ringqvist E. Troell K. Svärd S. G. (2010). Behind the Smile: Cell Biology and Disease Mechanisms of Giardia Species. Nat. Rev. Microbiol. 8 (6), 413–422. doi: 10.1038/nrmicro2317 20400969

[B9] Autheman D. Crosnier C. Clare S. Goulding D. A. Brandt C. Harcourt K.. (2021). An Invariant Trypanosoma Vivax Vaccine Antigen Induces Protective Immunity. Nature 595, 96–100. doi: 10.1038/s41586-021-03597-x 34040257

[B10] Bae S. S. Kim J. Kim T. S. Yong T. S. Park S. J. (2009). Giardia Lamblia: Immunogenicity and Intracellular Distribution of GHSP-115, a Member of the Giardia Head-Stalk Family of Proteins. Exp. Parasitol. 122 (1), 11–16. doi: 10.1016/j.exppara.2009.01.005 19545528

[B11] Belosevic M. Faubert G. M. Skamene E. MacLean J. D. (1984). Susceptibility and Resistance of Inbred Mice to Giardia Muris. Infect. Immun. 44 (2), 282–286. doi: 10.1128/iai.44.2.282-286.1984 6715033 PMC263514

[B12] Bevaart L. Van Ojik H. H. Sun A. W. Sulahian T. H. Leusen J. H.W. Weiner G. J.. (2004). CpG Oligodeoxynucleotides Enhance Fcγri-Mediated Cross Presentation by Dendritic Cells. Int. Immunol. 16 (8), 1091–1098. doi: 10.1093/intimm/dxh110 15192052

[B13] Bienz M. Dai W. J. Welle M. Gottstein B. Müller N. (2003). Interleukin-6-Deficient Mice Are Highly Susceptible to Giardia Lamblia Infection But Exhibit Normal Intestinal Immunoglobulin A Responses Against the Parasite. Infect. Immun. 71 (3), 1569–1573. doi: 10.1128/IAI.71.3.1569-1573.2003 12595479 PMC148820

[B14] Bienz M. Wittwer P. Zimmermann V. Müller N. (2001). Molecular Characterisation of a Predominant Antigenic Region of Giardia Lamblia Variant Surface Protein H7. Int. J. Parasitol. 31 (8), 827–832. doi: 10.1016/S0020-7519(01)00182-5 11403775

[B15] Borst P. Ulbert S. (2001). Control of VSG Gene Expression Sites. Mol. Biochem. Parasitol. 114, 17–27. doi: 10.1016/S0166-6851(01)00243-2 11356510

[B16] Cabrera-Licona A. Solano-González E. Fonseca-Liñán R. Bazán-Tejeda M. L. Argüello-García R. Bermúdez-Cruz R. M.. (2017). Expression and Secretion of the Giardia Duodenalis Variant Surface Protein 9B10A by Transfected Trophozoites Causes Damage to Epithelial Cell Monolayers Mediated by Protease Activity. Exp. Parasitol. 179, 49–64. doi: 10.1016/j.exppara.2017.06.006 28668253

[B17] Carranza P. G. Feltes G. Ropolo A. Quintana S. M.C. Touz M. C. Luján H. D. (2002). Simultaneous Expression of Different Variant-Specific Surface Proteins in Single Giardia Lamblia Trophozoites During Encystation. Infect. Immun. 70 (9), 5265–5268. doi: 10.1128/IAI.70.9.5265-5268.2002 12183579 PMC128263

[B18] Cecilio P. Pérez-Cabezas B. Fernández L. Moreno J. Carrillo E. Requena J. M.. (2017). Pre-Clinical Antigenicity Studies of an Innovative Multivalent Vaccine for Human Visceral Leishmaniasis. PloS Negl. Trop. Dis. 11 (11), e0005951. doi: 10.1371/journal.pntd.0005951 29176865 PMC5720812

[B19] Cedillo-Rivera R. Leal Y. A. Yépez-Mulia L. Gómez-Delgado A. Ortega-Pierres G. Tapia-Conyer R.. (2009). Seroepidemiology of Giardiasis in Mexico. Am. J. Trop. Med. Hyg. 80 (1), 6–10. doi: 10.4269/ajtmh.2009.80.6 19141830

[B20] Cotton J. Amat C. Buret A. (2015). Disruptions of Host Immunity and Inflammation by Giardia Duodenalis: Potential Consequences for Co-Infections in the Gastro-Intestinal Tract. Pathogens 4 (4), 764–792. doi: 10.3390/pathogens4040764 26569316 PMC4693164

[B21] Cotton J. A. Beatty J. K. Buret A. G. (2011). Host Parasite Interactions and Pathophysiology in Giardia Infections. Int. J. Parasitol. 41 (9), 925–933. doi: 10.1016/j.ijpara.2011.05.002 21683702

[B22] Crumpton M. J. (1974). “CHAPTER 1 - Protein Antigens: The Molecular Bases of Antigenicity and Immunogenicity,”. The antigens. Academic Press, p. 1–78. doi: 10.1016/B978-0-12-635502-4.50008-4.

[B23] Dann S. M. Manthey C. F. Le C. Miyamoto Y. Gima L. Abrahim A.. (2015). IL-17A Promotes Protective IgA Responses and Expression of Other Potential Effectors Against the Lumen-Dwelling Enteric Parasite Giardia. Exp. Parasitol. 156, 68–78. doi: 10.1016/j.exppara.2015.06.003 26071205 PMC4547885

[B24] Davids B. J. Liu C. M. Hanson E. M. Le C. H.Y. Ang J. Hanevik K.. (2019). Identification of Conserved Candidate Vaccine Antigens in the Surface Proteome of Giardia Lamblia. Infect. Immun. 87 (6), 1–14. doi: 10.1128/IAI.00219-19 PMC6529650 30962402

[B25] Davids B. J. Palm J.E.D. Housley M. P. Smith J. R. Andersen Y. S. Martin M. G.. (2006). Polymeric Immunoglobulin Receptor in Intestinal Immune Defense Against the Lumen-Dwelling Protozoan Parasite Giardia. J. Immunol. 177 (9), 6281–6290. doi: 10.4049/jimmunol.177.9.6281 17056558

[B26] Dissanayake S. K. Tuera N. Ostrand-Rosenberg S. (2005). Presentation of Endogenously Synthesized MHC Class II-Restricted Epitopes by MHC Class II Cancer Vaccines Is Independent of Transporter Associated With Ag Processing and the Proteasome. J. Immunol. 174 (4), 1811–1819. doi: 10.4049/jimmunol.174.4.1811 15699107

[B27] Dreesen L. De Bosscher K. Grit G. Staels B. Lubberts E. Bauge E.. (2014). Giardia Muris Infection in Mice Is Associated With a Protective Interleukin 17a Response and Induction of Peroxisome Proliferator-Activated Receptor Alpha. Infect. Immun. 82 (8), 3333–3340. doi: 10.1128/IAI.01536-14 24866800 PMC4136230

[B28] Eckmann L. (2003). Mucosal Defences Against Giardia. Parasite Immunol 25, 259–270. doi: 10.1046/j.1365-3024.2003.00634.x 12969444

[B29] El-Beshbishi S. Elblihy A. Atia R. Megahed A. Auf F. (2020). Human Leukocyte Antigen Class-II DRB1 Alleles and Giardia Lamblia Infection in Children: A Case-Control Study. Asian Pac. J. Trop. Med. 13 (2), 56–61. doi: 10.4103/1995-7645.275413

[B30] El-Manzalawy Y. Dobbs D. Honavar V. (2008). Predicting Linear B-Cell Epitopes Using String Kernels. J. Mol. Recognit. 21 (4), 243–255. doi: 10.1002/jmr.893 18496882 PMC2683948

[B31] Feliziani C. Merino M. C. Rivero M. R. Hellman U. Pistoresi-Palencia M. C. Rápolo A. S. (2011). Immunodominant Proteins α-1 Giardin and β-Giardin Are Expressed in Both Assemblages A and B of Giardia Lamblia. BMC Microbiol. 11 (1), 233. doi: 10.1186/1471-2180-11-233 22011206 PMC3206439

[B32] Feng X. M. Zheng W. Y. Zhang H. M. Shi W. Y. Li Y. Cui B. J.. (2016). Vaccination With Bivalent DNA Vaccine of A1-Giardin and Cwp2 Delivered by Attenuated Salmonella Typhimurium Reduces Trophozoites and Cysts in the Feces of Mice Infected With Giardia Lamblia. PloS One 11 (6), 1–16. doi: 10.1371/journal.pone.0157872 PMC4917239 27332547

[B33] Foged C. Brodin B. Frokjaer S. Sundblad A. (2005). Particle Size and Surface Charge Affect Particle Uptake by Human Dendritic Cells in an * In vitro* Model. Int. J. Pharm. 298 (2), 315–322. doi: 10.1016/j.ijpharm.2005.03.035 15961266

[B34] Franzén O. Jerlström-Hultqvist J. Castro E. Sherwood E. Ankarklev J. Reiner D. S.. (2009). Draft Genome Sequencing of Giardia Intestinalis Assemblage B Isolate GS: Is Human Giardiasis Caused by Two Different Species? PloS Pathog. 5 (8), e1000560. doi: 10.1371/journal.ppat.1000560. 19696920 PMC2723961

[B35] Garzon T. Valencia L. Dominguez V. Rascon L. Quintero J. Garibay-Escobar A.. (2020). Differential Antibody Responses to Giardia Lamblia Strain Variants Expressing Dissimilar Levels of an Immunogenic Protein. Parasite Immunol. 42 (10), 1–11. doi: 10.1111/pim.12767 32594543

[B36] Goodswen S. J. Kennedy P. J. Ellis J. T. (2017). On the Application of Reverse Vaccinology to Parasitic Diseases: A Perspective on Feature Selection and Ranking of Vaccine Candidates. Int. J. Parasitol. 47 (12), 779–790. doi: 10.1016/j.ijpara.2017.08.004 28893639

[B37] Greenbaum J. Sidney J. Chung J. Brander C. Peters B. Sette A. (2011). Functional Classification of Class II Human Leukocyte Antigen (HLA) Molecules Reveals Seven Different Supertypes and a Surprising Degree of Repertoire Sharing Across Supertypes. Immunogenetics 63 (6), 325–335. doi: 10.1007/s00251-011-0513-0 21305276 PMC3626422

[B38] Grit G. H. Van Coppernolle S. Devriendt B. Geurden T. Dreesen L. Hope J.. (2014). Evaluation of Cellular and Humoral Systemic Immune Response Against Giardia Duodenalis Infection in Cattle. Vet. Parasitol. 202 (3–4), 145–155. doi: 10.1016/j.vetpar.2014.03.012 24702771

[B39] Gupta S. Ansari H. R. Gautam A. Raghava G. P.S. (2013). Identification of B-Cell Epitopes in an Antigen for Inducing Specific Class of Antibodies. Biol. Direct 8 (1), 1–15. doi: 10.1186/1745-6150-8-27 24168386 PMC3831251

[B40] Hanevik K. Kristoffersen E. Svard S. Bruserud O. Ringqvist E. Sørnes S.. (2011). Human Cellular Immune Response Against Giardia Lamblia 5 Years After Acute Giardiasis. J. Infect. Dis. 204 (11), 1779–1786. doi: 10.1093/infdis/jir639 21990423

[B41] Hehl A. B. Marti M. Köhler P. (2000). Stage-Specific Expression and Targeting of Cyst Wall Protein-Green Fluorescent Protein Chimeras in Giardia. Mol. Biol. Cell 11 (5), 1789–1800. doi: 10.1091/mbc.11.5.1789 10793152 PMC14884

[B42] He Y. Xiang Z. Mobley H. L.T. (2010). Vaxign: The First Web-Based Vaccine Design Program for Reverse Vaccinology and Applications for Vaccine Development. J. Biomed. Biotechnol. 2010, 15. doi: 10.1155/2010/297505 PMC2910479 20671958

[B43] Heyworth M. F. Carlson J. R. Ermak T. H. (1987). Clearance of Giardia Muris Infection Requires Helper/Inducer T Lymphocytes. J. Exp. Med. 165 (6), 1743–1748. doi: 10.1084/jem.165.6.1743 2953846 PMC2188375

[B44] Hjøllo T. Bratland E. Steinsland H. Radunovic M. Langeland N. Hanevik K. (2018). Longitudinal Cohort Study of Serum Antibody Responses Towards Giardia Lamblia Variant-Specific Surface Proteins in a Non-Endemic Area. Exp. Parasitol. 191 (May), 66–72. doi: 10.1016/j.exppara.2018.06.005 29908864

[B45] Jenikova G. Hruz P. Andersson M. K. Tejman-Yarden N. Ferreira P. C.D. Andersen Y. S.. (2011). A1-Giardin Based Live Heterologous Vaccine Protects Against Giardia Lamblia Infection in a Murine Model. Vaccine 29 (51), 9529–9537. doi: 10.1016/j.vaccine.2011.09.126 22001876 PMC4045459

[B46] Jiménez-Cardoso E. Eligio-García L. Cortés-Campos A. (2002). Evaluación De La Capacidad Inmunogénica De La Vacuna Giardia-Vax, Utilizando Un Modelo Experimental De Giardiasis En Gerbos (Meriones Unguiculatus). Vet. Mex. 33 (1), 49–54. doi: 10.21753/vmoa.33.001.60

[B47] Jiménez J. C. Fontaine J. Creusy C. Fleurisse L. Grzych J. M. Capron M.. (2014). Antibody and Cytokine Responses to Giardia Excretory/Secretory Proteins in Giardia Intestinalis-Infected BALB/c Mice. Parasitol. Res. 113 (7), 2709–2718. doi: 10.1007/s00436-014-3927-4 24867815

[B48] Jiskoot W. Kersten G. F.A. Mastrobattista E. Slütter B. (2019). “Chapter 22-Vaccines,” in Pharmaceutical Biotechnology: Fundamentals and Applications. Eds. Crommelin D. J. A. Sindelar R. D. Meibohm B., Springer. 281–304.

[B49] Kamda J. D. Nash T. E. Singer S. M. (2012). Giardia Duodenalis: Dendritic Cell Defects in IL-6 Deficient Mice Contribute to Susceptibility to Intestinal Infection. Exp. Parasitol. 130 (3), 288–291. doi: 10.1016/j.exppara.2012.01.003 22248985 PMC3289762

[B50] Karosiene E. Rasmussen M. Blicher T. Lund O. Buus S. Nielsen M. (2013). NetMHCIIpan-3.0, a Common Pan-Specific MHC Class II Prediction Method Including All Three Human MHC Class II Isotypes, HLA-DR, HLA-DP and HLA-DQ. Immunogenetics 65 (10), 711–724. doi: 10.1007/s00251-013-0720-y 23900783 PMC3809066

[B51] Kaufmann S. H.E. Hess J. (1999). Impact of Intracellular Location of and Antigen Display by Intracellular Bacteria: Implications for Vaccine Development. Immunol. Lett. 65 (1–2), 81–84. doi: 10.1016/S0165-2478(98)00128-X 10065631

[B52] Kyes S. A. Kraemer S. M. Smith J. D. (2007). Antigenic Variation in Plasmodium Falciparum: Gene Organization and Regulation of the Var Multigene Family. Eukaryot. Cell 6, 1511–1520. doi: 10.1128/EC.00173-07 17644655 PMC2043368

[B53] Landais E. Romagnoli P. A. Corper A. L. Shires J. Altman J. D. Wilson I. A.. (2009). New Design of MHC Class II Tetramers to Accommodate Fundamental Principles of Antigen Presentation. J. Immunol. 183 (12), 7949–7957. doi: 10.4049/jimmunol.0902493 19923463 PMC2795019

[B54] Larocque R. Nakagaki K. Lee P. Abdul-Wahid A. Faubert G. M. (2003). Oral Immunization of BALB/c Mice With Giardia Duodenalis Recombinant Cyst Wall Protein Inhibits Shedding of Cysts. Infect. Immun. 71 (10), 5662–5669. doi: 10.1128/IAI.71.10.5662-5669.2003 14500486 PMC201086

[B55] Laurens M. B. (2020). RTS,S/AS01 Vaccine (MosquirixTM): An Overview. Hum. Vaccin. Immunother. 16 (3), 480–489. doi: 10.1080/21645515.2019.1669415 31545128 PMC7227679

[B56] Lee P. Abdul-Wahid A. Faubert G. M. (2009). Comparison of the Local Immune Response Against Giardia Lamblia Cyst Wall Protein 2 Induced by Recombinant Lactococcus Lactis and Streptococcus Gordonii. Microbes Infect. 11 (1), 20–28. doi: 10.1016/j.micinf.2008.10.002 18992359

[B57] Lee H. Y. Kim J. Noh H. J. Kim H. P. Park S. J. (2014). Giardia Lamblia Binding Immunoglobulin Protein Triggers Maturation of Dendritic Cells *via* Activation of TLR4-MyD88-P38 and ERK1/2 MAPKs. Parasite Immunol. 36 (12), 627–646. doi: 10.1111/pim.12119 24871487

[B58] Lima T. S. Lodoen M. B. (2019). Mechanisms of Human Innate Immune Evasion by Toxoplasma Gondii. Front. Cell. Infect. Microbiol. 9 (MAR), 1–8. doi: 10.3389/fcimb.2019.00103 31041194 PMC6476913

[B59] Li E. Zhou P. Petrin Z. Singer S. M. (2004). Mast Cell-Dependent Control of Giardia Lamblia Infections in Mice. Infect. Immun. 72 (11), 6642–6649. doi: 10.1128/IAI.72.11.6642-6649.2004 15501797 PMC523017

[B60] Lopez-Romero G. Garzon T. Rascon R. Valdez A. Quintero J. Arvizu-Flores A. A.. (2017). Characterization of BIP Protein of G. Lamblia as a Potential Immunogen in a Mouse Infection Model. Immunobiology 222 (8–9), 884–891. doi: 10.1016/j.imbio.2017.05.008 28552268

[B61] Lopez-Romero G. Quintero J. Astiazarán-García H. Velazquez C. (2015). Host Defences Against Giardia Lamblia. Parasite Immunol. 37 (8), 394–406. doi: 10.1111/pim.12210 26072999

[B62] Lovitch S. B. Unanue E. R. (2005). Conformational Isomers of a Peptide-Class II Major Histocompatibility Complex. Immunol. Rev. 207, 293–313. doi: 10.1111/j.0105-2896.2005.00298.x 16181344

[B63] Lujan H. D. (2006). Giardia and Giardiasis | Giardia Y Giardiasis. Medicina 66 (1), 70–74. 16555733

[B64] Lujan H. D. (2011). Mechanisms of Adaptation in the Intestinal Parasite Giardia Lamblia. Essays Biochem. 51 (1), 177–191. doi: 10.1042/bse0510177 22023449

[B65] Lujan H. D. Mowatt M. R. Conrad J. T. Bowers B. Nash T. E. (1995). Identification of a Novel Giardia Lamblia Cyst Wall Protein With Leucine- Rich Repeats: Implications for Secretory Granule Formation and Protein Assembly Into the Cyst Wall. J. Biol. Chem. 270 (49), 29307–29313. doi: 10.1074/jbc.270.49.29307 7493963

[B66] Lujan H. D. Svard S. (2011). Giardia A Model Organism. Wien: Springer, vol. 2011, 31–402.

[B67] Ma’ayeh S. Y. Liu J. Peirasmaki D. Hörnaeus K. Lind S. B. Grabherr M.. (2017). Characterization of the Giardia Intestinalis Secretome During Interaction With Human Intestinal Epithelial Cells: The Impact on Host Cells. PLoS Negl. Trop. Dis. 11 (12), e0006120. doi: 10.1371/journal.pntd.0006120 29228011 PMC5739509

[B68] Malonis R. J. Lai J. R. Vergnolle O. (2020). Peptide-Based Vaccines: Current Progress and Future Challenges. Chem. Rev. 120 (6), 3210–3229. doi: 10.1021/acs.chemrev.9b00472 31804810 PMC7094793

[B69] McCaffery J.M. Faubert G. M. Gillin F. D. (1994). Giardia Lamblia: Traffic of a Trophozoite Variant Surface Protein and a Major Cyst Wall Epitope During Growth, Encystation, and Antigenic Switching. Exp. Parasitol. 79 (3), 236–249. doi: 10.1006/expr.1994.1087 7525336

[B70] McFarland B. J. Sant A. J. Lybrand T. P. Beeson C. (1999). Ovalbumin(323-339) Peptide Binds to the Major Histocompatibility Complex Class II I-A(d) Protein Using Two Functionally Distinct Registers. Biochemistry 38 (50), 16663–16670. doi: 10.1021/bi991393l 10600129

[B71] Montagnoli C. Sandini S. Bacci A. Romani L. La Valle R. (2004). Immunogenicity and Protective Effect of Recombinant Enolase of Candida Albicans in a Murine Model of Systemic Candidiasis. Med. Mycol. 42 (4), 319–324. doi: 10.1080/13693780310001644653 15473356

[B72] Moormann A. M. Nixon C. E. Forconi C. S. (2019). Immune Effector Mechanisms in Malaria: An Update Focusing on Human Immunity. Parasite Immunol. 41 (8), 1–14. doi: 10.1111/pim.12628 30972776

[B73] Mora M. Telford J. L. (2010). Genome-Based Approaches to Vaccine Development. J. Mol. Med. 88 (2), 143–147. doi: 10.1007/s00109-009-0574-9 20066390

[B74] Mukherjee S. Huda S. Sinha Babu S. P. (2019). Toll-Like Receptor Polymorphism in Host Immune Response to Infectious Diseases: A Review. Scand. J. Immunol. 90 (1), 1–18. doi: 10.1111/sji.12771 31054156

[B75] Nash T. E. Aggarwal A. (1986). Cytotoxicity of Monoclonal Antibodies to a Subset of Giardia Isolates. J. Immunol. 136 (7), 2628 LP–2632. 3950421

[B76] Nash T. E. Merritt J. W. Conrad J. T. (1991). Isolate and Epitope Variability in Susceptibility of Giardia Lamblia to Intestinal Proteases. Infect. Immun. 59 (4), 1334–1340. doi: 10.1128/iai.59.4.1334-1340.1991 1706319 PMC257847

[B77] Nelson C. A. Roof R. W. McCourt D. W. Unanue E. R. (1992). Identification of the Naturally Processed Form of Hen Egg White Lysozyme Bound to the Murine Major Histocompatibility Complex Class II Molecule I-Ak. Proc. Natl. Acad. Sci. USA 89 (16), 7380–7383. doi: 10.1073/pnas.89.16.7380 1323833 PMC49713

[B78] Nielsen M. Lundegaard C. Blicher T. Peters B. Sette A. Justesen S.. (2008). Quantitative Predictions of Peptide Binding to Any HLA-DR Molecule of Known Sequence: NetMHCIIpan. PloS Comput. Biol. 4 (7), 1–10. doi: 10.1371/journal.pcbi.1000107 PMC2430535 18604266

[B79] Olson M. E. Ceri H. Morck D. W. (2000). Giardia Vaccination. Parasitol. Today 16 (5), 213–217. doi: 10.1016/S0169-4758(99)01623-3 10782082

[B80] Palm J.E.D. Weiland M. E.-L. Griffiths W. J. Ljungström I. Svärd S. G. (2003). Identification of Immunoreactive Proteins During Acute Human Giardiasis. J. Infect. Dis. 187 (12), 1849–1859. doi: 10.1086/375356 12792861

[B81] Papanastasiou P. Bruderer T. Li Y. Bommeli C. Köhler P. (1997). Primary Structure and Biochemical Properties of a Variant-Specific Surface Protein of Giardia1Note: The Nucleotide Sequence Information Reported in This Paper Has Been Submitted to the EMBL Data Library With the Accession No. Z83743.1. Mol. Biochem. Parasitol. 86 (1), 13–27. doi: 10.1016/S0166-6851(97)02836-3 9178264

[B82] Paul S. Sidney J. Sette A. Peters B. (2016). TepiTool: A Pipeline for Computational Prediction of T Cell Epitope Candidates. Curr. Protoc. Immunol. 114, 18.19.1–18.19.24. doi: 10.1002/cpim.12 27479659 PMC4981331

[B83] Pertsemlidis A. Fondon J. W. (2001). Having a BLAST With Bioinformatics (and Avoiding BLASTphemy). Genome Biol. 2 (10), reviews2002.1. doi: 10.1186/gb-2001-2-10-reviews2002 11597340 PMC138974

[B84] Potocnakova L. Bhide M. Borszekova Pulzova L. (2016). An Introduction to B-Cell Epitope Mapping and *In Silico* Epitope Prediction. J. Immunol. Res. 2016, 11. doi: 10.1155/2016/6760830 PMC5227168 28127568

[B85] Priest J. W. Mehlert A. Arrowood M. J. Riggs M. W. Ferguson M. A. J. (2003). Characterization of a Low Molecular Weight Glycolipid Antigen From Cryptosporidium Parvum. J. Biol. Chem. 278 (52), 52212–52222. doi: 10.1074/jbc.M306835200 14557271

[B86] Quintero J. Valdez A. Samaniego B. Lopez-Romero G. Astiazaran-Garcia H. Rascon L.. (2017). Isolation and Partial Characterization of an Immunogenic Antigen of Giardia Lamblia. Parasitol. Int. 66 (3), 324–330. doi: 10.1016/j.parint.2017.01.007 28110081

[B87] Radunovic M. Klotz C. Saghaug C. S. Brattbakk H. R. Aebischer T. Langeland N.. (2017). Genetic Variation in Potential Giardia Vaccine Candidates Cyst Wall Protein 2 and A1-Giardin. Parasitol. Res. 116 (8), 2151–2158. doi: 10.1007/s00436-017-5516-9 28578460

[B88] Reiner D. S. Gillin F. D. (1991). Human Secretory and Serum Antibodies Recognize Environmentally Induced Antigens of Giardia Lamblia. Infect. Immun. 60 (2), 637–643. doi: 10.1128/iai.60.2.637-643.1992 PMC257677 1730497

[B89] Rhee J. H. Lee S. E. Kim S. Y. (2012). Mucosal Vaccine Adjuvants Update. Clin. Exp. Vaccine Res. 1 (1), 50–63. doi: 10.7774/cevr.2012.1.1.50 23596577 PMC3623511

[B90] Rivero F. D. Saura A. Prucca C. G. Carranza P. G. Torri A. Lujan H. D. (2010). Disruption of Antigenic Variation Is Crucial for Effective Parasite Vaccine. Nat. Med. 16 (5), 551–557. doi: 10.1038/nm.2141 20418884

[B91] Robleda-Castillo R. Ros-Lucas A. Martinez-Peinado N. Alonso-Padilla J. (2021). An Overview of Current Uses and Future Opportunities for Computer-Assisted Design of Vaccines for Neglected Tropical Diseases. Adv. Appl. Bioinform. Chem. 14, 25–47. doi: 10.2147/AABC.S258759 33623396 PMC7894434

[B92] Ropert C. Gazzinelli R. T. (2000). Signaling of Immune System Cells by Glycosylphosphatidylinositol (GPI) Anchor and Related Structures Derived From Parasitic Protozoa. Curr. Opin. Microbiol. 3 (4), 395–403. doi: 10.1016/S1369-5274(00)00111-9 10972501

[B93] Roxström-Lindquist K. Ringqvist E. Palm D. Svärd S. (2005). Giardia Lamblia-Induced Changes in Gene Expression in Differentiated Caco-2 Human Intestinal Epithelial Cells. Infect. Immun. 73 (12), 8204–8208. doi: 10.1128/IAI.73.12.8204-8208.2005 16299316 PMC1307045

[B94] Saghaug C. S. Sørnes S. Peirasmaki D. Svärd S. Langeland N. Hanevik K. (2015). Human Memory CD4+ T Cell Immune Responses Against Giardia Lamblia. Clin. Vaccine Immunol. 23 (1), 11–18. doi: 10.1128/CVI.00419-15 26376930 PMC4711086

[B95] Scott K. G.E. Yu L. C.H. Buret A. G. (2004). Role of CD8+ and CD4+ T Lymphocytes in Jejunal Mucosal Injury During Murine Giardiasis. Infect. Immun. 72 (6), 3536–3542. doi: 10.1128/IAI.72.6.3536-3542.2004 15155662 PMC415705

[B96] Serradell M. C. Gargantini P. R. Saura A. Oms S. R. Rupil L. L. Berod L.. (2018). Cytokines, Antibodies, and Histopathological Profiles During Giardia Infection and Variant-Specific Surface Protein-Based Vaccination. Infect. Immun. 86 (6), e00773–e00717. doi: 10.1128/IAI.00773-17 29555679 PMC5964528

[B97] Serradell M. C. Rupil L. L. Martino R. A. Prucca C. G. Carranza P. G. Saura A.. (2019). Efficient Oral Vaccination by Bioengineering Virus-Like Particles With Protozoan Surface Proteins. Nat. Commun. 10 (1), 361. doi: 10.1038/s41467-018-08265-9 30664644 PMC6341118

[B98] Serradell M. C. Saura A. Rupil L. L. Gargantini P. R. Faya M. I. Furlan P. J.. (2016). Vaccination of Domestic Animals With a Novel Oral Vaccine Prevents Giardia Infections, Alleviates Signs of Giardiasis and Reduces Transmission to Humans. NPJ Vaccines 1, 16018. doi: 10.1038/npjvaccines.2016.18 29263857 PMC5707882

[B99] Shimoda M. Koni P. A. (2007). MHC-Restricted B-Cell Antigen Presentation in Memory B-Cell Maintenance and Differentiation. Crit. Rev. Immunol. 27 (1), 47–60. doi: 10.1615/CritRevImmunol.v27.i1.40 17430096

[B100] Singer S. M. (2016). Control of Giardiasis by Interleukin-17 in Humans and Mice - Are the Questions All Answered? Clin. Vaccine Immunol. 23 (1), 2–5. doi: 10.1128/CVI.00648-15 26581888 PMC4711094

[B101] Singer S. M. Nash T. E. (2000). T-Cell-Dependent Control of Acute Giardia Lamblia Infections in Mice. Infect. Immun. 68 (1), 170–175. doi: 10.1128/IAI.68.1.170-175.2000 10603384 PMC97117

[B102] Skwarczynski M. Toth I. (2016). Peptide-Based Synthetic Vaccines. Chem. Sci. 7 (2), 842–854. doi: 10.1039/C5SC03892H 28791117 PMC5529997

[B103] Smith N. C. Sinden R. E. Ramakrishnan C. (2021). Editorial: Get Over the Gut: Apicomplexan Parasite Interaction, Survival and Stage Progression in Vertebrate and Invertebrate Digestive Tracts 11, 680555. doi: 10.3389/fcimb.2021.680555 PMC8113612 33996642

[B104] Stäger S. Felleisen R. Gottstein B. Müller N. (1997). Giardia Lamblia Variant Surface Protein H7 Stimulates a Heterogeneous Repertoire of Antibodies Displaying Differential Cytological Effects on the Parasite. Mol. Biochem. Parasitol. 85 (1), 113–124. doi: 10.1016/S0166-6851(96)02818-6 9108553

[B105] Stäger S. Gottstein B. Sager H. Jungi T. W. Müller N. (1998). Influence of Antibodies in Mother’s Milk on Antigenic Variation of Giardia Lamblia in the Murine Mother-Offspring Model of Infection. Infect. Immun. 66 (4), 1287–1292. doi: 10.1128/IAI.66.4.1287-1292.1998 9529044 PMC108051

[B106] Stein J. E. Radecki S. V. Lappin M. R. (2003). Efficacy of Giardia Vaccination in the Treatment of Giardiasis in Cats. J. Am. Vet. Med. Assoc. 222 (11), 1548–1551. doi: 10.2460/javma.2003.222.1548 12784960

[B107] Strong B. S.I. Unanue E. R. (2011). Presentation of Type B Peptide-MHC Complexes From Hen Egg White Lysozyme by TLR Ligands and Type I IFNs Independent of H2-DM Regulation. J. Immunol. (Baltimore Md.: 1950) 187 (5), 2193–2201. doi: 10.4049/jimmunol.1100152 PMC3509772 21788443

[B108] Sun C. H. J. McCaffery M. Reiner D. S. Gillin F. D. (2003). Mining the Giardia Lamblia Genome for New Cyst Wall Proteins. J. Biol. Chem. 278 (24), 21701–21708. doi: 10.1074/jbc.M302023200 12686559

[B109] Tedla M. G. Every A. L. Scheerlinck J. P. Y. (2019). Investigating Immune Responses to Parasites Using Transgenesis. Parasit. Vectors 12 (1), 1–14. doi: 10.1186/s13071-019-3550-4 31202271 PMC6570953

[B110] Teh-Poot C. Tzec-Arjona E. Martínez-Vega P. Jesus Ramirez-Sierra M. Rosado-Vallado M. Dumonteil E. (2015). From Genome Screening to Creation of Vaccine Against Trypanosoma Cruzi by Use of Immunoinformatics. J. Infect. Dis. 211 (2), 258–266. doi: 10.1093/infdis/jiu418 25070943

[B111] Téllez A. Palm D. Weiland M. Alemán J. Winiecka-Krusnell J. Linder E.. (2005). Secretory Antibodies Against Giardia Intestinalis in Lactating Nicaraguan Women. Parasite Immunol. 27 (5), 163–169. doi: 10.1111/j.1365-3024.2005.00758.x 15987339

[B112] Troeger H. Joerg Epple H. Schneider T. Wahnschaffe U. Ullrich R. Burchard G. D.. (2007). Effect of Chronic Giardia Lamblia Infection on Epithelial Transport and Barrier Function in Human Duodenum. Gut 56 (3), 328–335. doi: 10.1136/gut.2006.100198 16935925 PMC1856804

[B113] Uwase J. Chu R. Kassegne K. Lei Y. Shen F. Fu H.. (2020). Immunogenicity Analysis of Conserved Fragments in Plasmodium Ovale Species Merozoite Surface Protein 4. Malaria J. 19 (1), 1–11. doi: 10.1186/s12936-020-03207-7 PMC7106901 32228600

[B114] Vakili B. Nezafat N. Zare B. Erfani N. Akbari M. Ghasemi Y.. (2020). A New Multi-Epitope Peptide Vaccine Induces Immune Responses and Protection Against Leishmania Infantum in BALB/c Mice. Med. Microbiol. Immunol. 209 (1), 69–79. doi: 10.1007/s00430-019-00640-7 31696313

[B115] Velazquez C. Beltran M. Ontiveros N. Rascon L. Figueroa D. C. Granados A. J.. (2005). Giardia Lamblia Infection Induces Different Secretory and Systemic Antibody Responses in Mice. Parasite Immunol. 27 (9), 351–356. doi: 10.1111/j.1365-3024.2005.00793.x 16149993

[B116] Velazquez C. Vidavsky I. Der Van Drift K. Gross M. L. Unanue E. R. (2002). Chemical Identification of a Low Abundance Lysozyme Peptide Family Bound to I-A K Histocompatibility Molecules. J. Biol. Chem. 277 (45), 42514–42522. doi: 10.1074/jbc.M202316200 12055186

[B117] Venkatesan P. Finch R. G. Wakelin D. (1997). A Comparison of Mucosal Inflammatory Responses to Giardia Muris in Resistant B10 and Susceptible BALB/c Mice. Parasite Immunol. 19 (3), 137–143. doi: 10.1046/j.1365-3024.1997.d01-189.x 9106819

[B118] Wang P. Sidney J. Dow C. Mothé B. Sette A. Peters B. (2008). A Systematic Assessment of MHC Class II Peptide Binding Predictions and Evaluation of a Consensus Approach. PloS Comput. Biol. 4 (4), e1000048. doi: 10.1371/journal.pcbi.1000048 18389056 PMC2267221

[B119] Wang P. Sidney J. Kim Y. Sette A. Lund O. Nielsen M.. (2010). Peptide Binding Predictions for HLA DR, DP and DQ Molecules. BMC Bioinformatics 11 (1), 568. doi: 10.1186/1471-2105-11-568 21092157 PMC2998531

[B120] Wang Y. Yi L. Sun L. Y. Liu Y. C. Wen W. Y. Li X. K.. (2020). Identification and Characterization of a Streptococcus Suis Immunogenic Ornithine Carbamoytransferase Involved in Bacterial Adherence. J. Microbiol. Immunol. Infect. 53 (2), 234–239. doi: 10.1016/j.jmii.2018.05.004 29934035

[B121] Weaver C. T. Harrington L. E. Mangan P. R. Gavrieli M. Murphy K. M. (2006). Th17: An Effector CD4 T Cell Lineage With Regulatory T Cell Ties. Immunity 24 (6), 677–688. doi: 10.1016/j.immuni.2006.06.002 16782025

[B122] Weiland M. E.L. Palm J.E.D. Griffiths W. J. McCaffery J.M. Svärd S. G. (2003). Characterisation of Alpha-1 Giardin: An Immunodominant Giardia Lamblia Annexin With Glycosaminoglycan-Binding Activity. Int. J. Parasitol. 33 (12), 1341–1351. doi: 10.1016/S0020-7519(03)00201-7 14527517

[B123] Wong-Baeza I. Alcántara-Hernández M. Mancilla-Herrera I. Ramírez-Saldívar I. Arriaga-Pizano L. Ferat-Osorio E.. (2010). The Role of Lipopeptidophosphoglycan in the Immune Response to Entamoeba Histolytica. J. Biomed. Biotechnol. 2010, 254521. doi: 10.1155/2010/254521 20145703 PMC2817369

[B124] Xiang Z. He Y. (2009). Vaxign: A Web-Based Vaccine Target Design Program for Reverse Vaccinology. Proc. Vaccinol. 1 (1), 23–29. doi: 10.1016/j.provac.2009.07.005

