# Supplementary material for: “Immunoinformatic Identification of T-Cell and B-Cell Epitopes From Giardia lamblia Immunogenic Proteins as Candidates to Develop Peptide-Based Vaccines Against Giardiasis”
Source: Front Cell Infect Microbiol. 2021 Oct 27;11:769446. doi: 10.3389/fcimb.2021.769446 (PMC8579046; doi:10.3389/fcimb.2021.769446)
Supplement: Supplementary file 1 [file DataSheet_1.pdf]

Table S1. Murine MHC class II binding sequences prediction.

		MHC class II IA^k					MHC class II IA^d				
	**Protein**	**Position**	**Epitope**	**Assemblages**	**Affinity (nM)**	**%Rank**	**Position**	**Epitope**	**Assemblages**	**Affinity (nM)**	**%Rank**
Structural proteins	α-1-giardin	173	YLIDFFGTVPSAEYR	A	6809.93	0.60	34	SREKIAKAYVASYGK	A	162.26	1.70
		175	IDFFGTVPSAEYRPI		7549.11	1.20	280	FDKRMAPILRTLWRV		188.66	2.50
		187	RPIAEAFKAQNGKS		7778.11	1.50	222	FALLGMHRLAAYLIN		214.88	3.00
		21	VQIAFIASEYSAESR		7737.23	1.50	246	KRMRRITGMMVDKCL		230.21	3.50
		183	SAEYRPIAEAFKAQN		8782.92	3.50	224	LLGMHRLAAYLINCA		275.27	4.50
	α-2-giardin	173	YISSFMAGVPPEEYK	A	4529.08	0.02	22	FALLGMHKLAAYLVN	A	187.85	2.50
			YLIDFFGTVPSAEYR	B	6809.93	0.60					
		176	SFMAGVPPEEYKSIN	A	6227.85	0.30	280	FDKRMAPILRTLWRV	B	188.66	2.50
		263	KYAYKTYGSMKADVE	A	6856.88	0.60	222	FALLGMHRLAAYLIN	B	214.88	3.00
		24	AFIASEYSADARQRV	B	7501.8	1.20	246	KRMRRITGMMVDKCL	A/B	230.21	3.50
			AFIASEYSADARQRI	A	7531.4	1.20					
		21	GQIAFIASEYSADAR	B	7630.65	1.30	224	LLGMHRLAAYLINCA		275.27	4.50
			AQIAFIASEYSADAR	A	7807.2	1.50					
	α-7.1-giardin	14	QHLLRGATAQAAGRA	A	5456.24	0.09	14	QHLLRGATAQAAGRA	A	42.4	0.04
		124	RQRAEIYAAFRAANG		7071.31	0.80	89	SAKLKMAAAKATEIK		45.63	0.06
		121	FDDRQRAEIYAAFRA		7574.63	1.30	91	KLKMAAAKATEIKAL		57.02	0.12
		91	KLKMAAAKATEIKAL		7785.35	1.50	86	RNSSAKLKMAAAKAT		79.17	0.40
		129	IYAAFRAANGKTASE		8476.32	3.00	126	RAEIYAAFRAANGKT		116.38	0.90
	α-7.3-giardin	31	KQRAEIHAAFRAATG	A	6319.49	0.40	31	KQRAEIHAAFRAATG	A	118.98	0.90
		32	QRAEIHAAFRAATGK		7113.74	0.90	33	RAEIHAAFRAATGKT		123.89	1.00
		28	FNDKQRAEIHAAFRA		7649.17	1.30	34	AEIHAAFRAATGKTT		181.18	2.50
		184	SEWKQISEAFENIAK		9426.62	6.0	129	GSKTVWARMVNSWLA		231.73	3.50
		263	KVLYAHYGNLAKDIR		10229.00	9.5	130	SKTVWARMVNSWLAF		233.8	3.50
	α-11-giardin	221	IAHYYNLAPARAVAY	A/B	3636.76	0.01	223	HFYNLAPARAVAYAF	B	40.47	0.03
		224	YYNLAPARAVAYAFH	A	5439.79	0.09		HYYNLAPARAVAYAF	A	43.07	0.05
			FYNLAPARAVAYAFY	B	5635.09	0.12	220	CIAHFYNLAPARAVA	B	118.46	0.90
		218	GLCIAHYYNLAPARA	A	7073.98	0.80		CIAHYYNLAPARAVA	A	136.82	1.20
			GLCIAHFYNLAPARA	B	8093.97	2.50	227	LAPARAVAYAFHSAV	A	149.23	1.50
		231	RAVAYAFHSAVETQN	A	8470.27	3.00		LAPARAVAYAFYSAV	B	186.67	2.50
		103	PDERLKLAQAYQEKT	B	8556.12	3.00	219	LCIAHYYNLAPARAV	A	238.04	3.50
							124	DFVDQIPLTSAASYL	A	238.26	3.50
	β-giardin	135	QIAIHNDAIAALRKE	A/B	6091.83	0.25	192	KETIARERAVSAATT	A/B	198.02	2.5
		133	QNQIAIHNDAIAALR		7198.29	0.90	178	KVAEGFARISAAIEK		244.45	3.5
		178	KVAEGFARISAAIEK		7364.28	1.10	195	IARERAVSAATTEAL		281.17	4.5
		180	AEGFARISAAIEKET		7707.90	1.40	181	EGFARISAAIEKETI		280.12	4.5
		104	KEEIDTMAANFRKSL		9470.37	6.00	258	VVSKIQGGLSMVTKH		330.97	5.5
	SALP-1	58	KQTLQRAEASHAMDK	A/B	7818.27	1.70	173	ESSKRAYHSVIASYK	A/B	171.36	1.90
		173	ESSKRAYHSVIASYK		7884.87	1.70					
		128	FRKEYGTAMEGLAEK		8040.64	1.90	163	KDFMRLIESIESSKR		240.23	3.50
		60	TLQRAEASHAMDKM	A	8101.94	2.50					

			LQRAEASHAMDKM	B	8101.94	2.50	56	IQKQTLQRAEASHAM		258.35	4.00
		129	RKEYGTAMEGLAEKL	A/B	8505.26	3.00					
	21.1 protein	322	TCKNYINALQSEKAK	B	7029.58	0.80	508	ELTKSSVALQALQNG	A/B	86.4	0.50
		692	GMTLENGWTALMSAV	A/B	7216.07	0.90	17	NHSAIRTAIPRFAGS		117.95	0.90
		482	ARRKAAYNALESKKE	A/B	7803.74	1.5	767	RRAIFYAKSAEVKNL	B	128.2	1.10
		636	PKTYLMSAAEHGELL	A	8132.07	2.5		RRAIFYAKSAEIKNL	A	156.69	1.60
		507	DELTKSSVALQALQN	A/B	8140.08	2.5	510	TKSSVALQALQNGIS	A/B	139.63	1.20
							65	PDNLTALMIAAQSDH		153.85	1.50
	α-Tubulin	290	ELTNSVFEPANMMVK	A/B	7593.26	1.30	326	KDVNAAIAVIKTKRT	A/B	76.23	0.30
		184	PYNTILAAHSMLEHS		7635.11	1.30	182	VEPYNTILAAHSMLE		92.23	0.50
		253	TEFQTNLVPYPRIHF		8225.34	2.50	185	YNTILAAHSMLEHSD		164.24	1.80
		166	KLEFVVYPSPQIATA		8277.29	2.50	230	LIAQCISSITASLRF		165.31	1.80
		266	HFPLCSYAPIISSEK		8443.82	3.00	373	RSCLMISNTTAIAEV		215.73	3.00
	β-Tubulin	272	PLTSRGSQIYRALTV	A	5938.82	0.20	306	RHGRYLTAAAMFRGR	A	80.25	0.40
		275	SRGSQIYRALTVPEL		7010.82	0.70	305	PRHGRYLTAAAMFRG		107.65	0.70
		304	DPRHGRYLTAAAMFR		8142.90	2.50	277	GSQIYRALTVPELVS		136.23	1.20
		339	SYFVEWIPNNMKVSV		8332.74	2.50	358	PRGLKMAATFIGNST		154.85	1.60
		161	DRMMCTFSVVPSPKV		8445.5	3.00	275	SRGSQIYRALTVPEL		209.05	3.00
	GHSP-115	354	LLNEAARALPPLSPY	A	4967.03	0.04	518	ELQAARAIAEAKLAA	A	72.82	0.25
		516	SDELQAARAIAEAKL		5886.75	0.17	519	LQAARAIAEAKLAAA		102.93	0.70
		308	PNGPAAAAAMLDAAS		7051.36	0.80	36	GETGLMKAVRSNNVD		126.66	1.00
		1004	EMERQAAAISYYQQL		7398.86	1.10	128	RGRTALMYAAEAGHL		147.79	1.40
		133	LMYAAEAGHLVIAEA		7716.25	1.40	96	KEQQTALMLAAGAGA		152.58	1.50
Metabolic Proteins	ADI	381	PTIDFIKASPAYISY	B	5149.2	0.05	381	PTIDFIKASPAYISY	B	52.57	0.09
			PTVDFIKADPAYISY	A	6470.04	0.40	380	QPTIDFIKASPAYIS		71.52	0.25
		101	KYEFHPSGARITPKM	A/B	6095.59	0.25	123	YKRKVLSALSTRNLV	A	78.29	0.40
		516	PYITWRMPSMPVVSL		6679.74	0.50		YKRKVLSALSNRNLV	B	105.84	0.70
		517	YITWRMPSMPVVSLK		6707.48	0.50	517	YITWRMPSMPVVSLK	A/B	105.66	0.70
		89	REVLMDQAMASLKYE		7210.3	0.90	520	WRMPSMPVVSLKGAA		124.71	1.00
	OCT	305	EAGNRLHSAMAVLDF	A/B	6882.45	0.70	4	KQTRHLLTISALSPK	B	80.11	0.40
		256	LKVLTPFQVDDAVMA		8199.92	2.50		KQTRHLLTISALCPK	A	153.92	1.50
			LKILTPFQVDDAVMA		8211.37	2.50	5	QTRHLLTISALSPKE	B	91.81	0.50
		34	PAKYTARAANKTLLA		8823.3	4.00	306	AGNRLHSAMAVLDFF	A/B	107.6	0.70
		7	RHLLTISALSPKELM	B	8866.37	4.00	8	HLLTISALSPKELMY	B	179.45	2.50
		17	PKELAYLIDRALDMK	A	8959.52	4.50	43	NKTLLAFFAKPSLRT	A/B	189.62	2.50
	FBA	122	VAYAHARGVSVEAEL	A/B	6757.59	0.60	258	SRMAMTGAIRKVFAE	B	107.31	0.70
		17	KYGVGAFNVNNMEQI		6814.57	0.60	257	DSRMAMTGAIRKVFV	A	112.21	0.80
		121	VVAYAHARGVSVEAE		7125.45	0.90	29	EQIQGIMKAVVQLKS	A/B	139.14	1.20
		257	DSRMAMTGAIRKVFA	B	9072.49	4.50	31	IQGIMKAVVQLKSPV		154.54	1.60
		20	VGAFNVNNMEQIQGI	A/B	9182.9	5.00	28	MEQIQGIMKAVVQLK		168.58	1.90
	UPL-1	236	AVHMSAAHIALAQR	B	5650.66	0.12	236	AVHMSAAHIALAQRK	B	35.06	0.02
			AVYMSAAHIALAQRK	A	6437.57	0.40		AVYMSAAHIALAQRK	A	40.3	0.03
		206	IKTVPGFSMMEMESA	A/B	6829.78	0.60	234	CGAVHMSAAHIALAQ	B	44.58	0.05
		238	YMSAAHIALAQRKSN	A	7075.44	0.80	159	SIVRKHVAALSYKMH		87.48	0.50
			HMSAAHIALAQRKSN	B	7331.6	1.10	161	VRKHVAALSYKMHAG		137.5	1.20

		205	YIKTVPGFSMMEMES	A/B	7433.04	1.20	239	MSAAHIALAQRKSND	A/B	140.28	1.30
		233	VCGAVHMSAAHIALA	B	7803.14	1.50	232	SVCGAVYMSAAHIAL	A	154.57	1.60
	Enolase	107	FSILGANAVLPVSMA	A/B	7192.76	0.90	2	EAPSTIKAIKARMII	A/B	40.24	0.03
		367	TITGAIDAANLAMSY		7477.73	1.20	1	MEAPSTIKAIKARMI		47	0.06
		366	GTITGAIDAANLAMS		7693.57	1.40	70	LENIRKIIAPALIGM		78.93	0.40
		220	GDEGGFAPNVADPEV		7754.58	1.50	107	0FSILGANAVLPVSMA		83.21	0.40
		365	VGTITGAIDAANLAM		7757.18	1.50	105	KTFSILGANAVLPVS		106.08	0.70
VSP	VSP9B10	123	QAAQGYFVPPGADAS	A	8046.21	1.90					
		51	SRCNTGFVPINGQCA		8094.84	2.50					
	TSA 417	119	GYFAPVGAANTEQSV		8571.68	3.00					
	VSP-5G8	116	KDGYFTVSDATATQD	B	8888.46	4.00	8	VAVILQIARAACTPG	B	107.82	0.70
							7	LVAVILQIARAACTP		112.92	0.80
							9	AVILQIARAACTPGT		113.5	0.80
	VSPH7	183	ANLYLKAVSSPTSAT		8965.15	4.50	182	NANLYLKAVSSPTSA		100.73	0.60
							184	NLYLKAVSSPTSATS		123.02	1.00
HSP	BIP	396/411	DEAVAWGAAVQASIL	A/B	6320.86	0.40	152/167	EKITKAVVTVPAYFS	A/B	70.8	0.25
		38	IPNELGARVTPSYVA	A	7027.22	0.80	397/412	EAVAWGAAVQASILS		176.23	2.00
		53	PNELGARVTPSYVA	B	7027.22	0.80	155/170	TKAVVTVPAYFSDSQ		185.65	2.50
		64/79	AAKNYAPISPENTIF	A/B	7722.09	1.40	300/315	GKRQLSTASSVQIVV		194.62	2.50
		64/79	AAKNYAPISPENTIF	A/B	7722.09	1.40	509/524	SNGILTVSAVEKSSG		212.26	3.00
		440/445	LIERNSYIPVKKSKI	A/B	7992.24	1.90	399/414	VAWGAAVQASILSGA		223.97	3.00
CWPs	CWP 1	91	YLSNNSLAGAIPEGL	A	6432.84	0.40	2	MLALLALAGSALALT	A	98.58	0.60
	CWP 2	91	YLNNNDLAGAIPTDL	B	8265.57	2.50	289	LRTVVRSSSSRASSA	B	109.38	0.80
	CWP 3	9	IEIGYGLADAQHDAL	A/B	6701.82	0.50	108	LTNLQYLQINKAGLT		186.71	2.50
	CWP 2	37	WKSNNWLAADVSYCS		7480.48	1.20	108	LTNLQYLQVNSAGLT	A	211.46	3.00
		68	LSDMGLTGALPADIG		7808.38	1.50	6	VLGLLGLARAACPAT		243.72	3.5
		321	GNASRSAVARPTARA		7872.77	1.70	111	LQYLQINKAGLTGSI		250.91	4.00
Giardia trophozoite antigen	GTA-1	100	LELIMSLAPNHMSAI	A/B	5487.86	0.10	100	LELIMSLAPNHMSAI		57.55	0.12
	GTA-2	92	NASYHCAAAFQDSIR	B	6668.76	0.50	20	VVNEIRATKVVMVSH	B	70.45	0.25
		95	YHCAAAFQDSIRPEF	B	7618.52	1.30	20	VVNEIRATKVVLVSH	A	95.99	0.60
		47	ICRKRGIEVYPVDVP	B	7777.02	1.50	124	GYYMYMGKTVHSDRA	A/B	174.01	2.00
		8	NRLYYRPSAFPTVVN	A/B	7960.82	1.80	103/ GTA1	IMSLAPNHMSAICTV		192.54	2.50

The epitopes shown are the top 5 binder sequences in MHC class II binding prediction of each protein.

It is considered strong binder > 2% rank and weak binder < 10% rank.
