# Supplementary material for: “Immunoinformatic Identification of T-Cell and B-Cell Epitopes From Giardia lamblia Immunogenic Proteins as Candidates to Develop Peptide-Based Vaccines Against Giardiasis”
Source: Front Cell Infect Microbiol. 2021 Oct 27;11:769446. doi: 10.3389/fcimb.2021.769446 (PMC8579046; doi:10.3389/fcimb.2021.769446)
Supplement: Supplementary file 2 [file DataSheet_2.pdf]

Table S2. HLA class II binding sequences prediction.

		HLA class-II DRB1*03:01					HLA class-II DRB1*13:01				
Protein		Position	Epitope	Assemblages	Affinity (nM)	%Rank	Position	Epitope	Assemblages	Affinity (nM)	%Rank
Structural proteins	α-1-giardin	65	SLLMDLFSDRHEVRA	A	123.11	2.5	244	DEKRMRRITGMMVDK	A	24.17	0.9
		250	RITGMMVDKCLGAKH		255.88	6	221	HFALLGMHRLAAYLI		24.69	1
		264	HAYKIYGDMGTDIER		344.9	8	281	DKRMAPILRTLWRVK		24.9	1
		281	DKRMAPILRTLWRVK		427.81	10	224	LLGMHRLAAYLINCA		47.24	4
							110	TVAAYTRMFKKPLVE		60.97	6.5
	α-2-giardin	4	LSQIVADIKQAIDAK	B	24.48	0.03	244	DEKRMRRITGMMVDK	A/B	24.17	0.9
			LSQIVADMKQAIDK	A							
		250	RITGMMVDKCLAAKY	A	113.93	2	221	HFALLGMHRLAAYLI	B	24.69	1
		65	SLLMDLFSDRHEVRA	A/B	123.11	2.5	281	DKRMAPILRTLWRVK	B	24.9	1
		64	ESLLMDLFSDRHEVR	A/B	157.89	3.5	221	HFALLGMHKLAAYLV	A	30.56	1.7
		26	IASEYSADARQRIAQ	A	178.3	4	224	LLGMHRLAAYLINCA	B	47.24	4
			IASEYSADARQRVAQ	B	179.03	4		LLGMHKLAAYLVNCA	A	54.33	5
	α-7.1-giardin	157	LMMIVLDDEIDVRCK	A	50.29	0.5	6	YGLAMIVGQHLLRGA	A	37.25	3
		361	HYGNLAKDIRATMSK		79.69	1.2	366	AKDIRATMSKNLAEA		50.19	4.5
							8	LAMIVGQHLLRGATA		54.08	5
		160	IVLDDEIDVRCKLIK		179.16	4	229	RMVNSWLAFCRSARN		71.65	8
							228	ARMVNSWLAFCRSAR		72.15	8
	α-7.3-giardin	64	LMMIVLDDEIDVRCR	A	48.53	0.4	273	AKDIRKTMSKNLAEA	A	51.22	5
		67	IVLDDEIDVRCRLIK		48.53	3	72	EIDVRCRLIKKAFKG		54.04	5
		268	HYGNLAKDIRKTMSK		163.48	3.5	138	VNSWLAFCRSARNNV		70.24	7.5
		68	VLDDEIDVRCRLIKK		392.59	9.5	136	RMVNSWLAFCRSARN		71.65	8
							32	QRAEIHAAFRAATGK		83.56	9.5
	α-11-giardin	274	WGVMRDDIISRFQSK	B	37.88	0.17	75	SARVNVIKKAMKNVN	A	19.64	0.5
			WGVMRDDILSRFQSK	A	44.39	0.3		SARVNVIKKAMKGVN	B	19.76	0.5
		96	DVILIATPDERLKLA	B	88.13	1.4	77	RVNVIKKAMKNVNDF	A	22.94	0.8
		97	VILIATPDERLKLAQ	B	88.98	1.4	78	VNVIKKAMKGVNDFR	B	34.47	2.5
			VVLIATPDERLKLAQ	A	107.14	1.9	223	HFYNLAPARAVAYAF	B	51.27	5
		27	VMRDDIISRFQSKDA	B	90.45	1.4		HYYNLAPARAVAYAF	A	56.12	5.5
			VMRDDILSRFQSKEA	A	120.83	2.5	71	DCNISARVNVIKKAM	A/B	62.67	6.5
	β-giardin	59	RVKMIKDAIAHLDRL	A/B	101.39	1.7	140	NDAIAALRKEALKSL	A/B	40.32	3.5
		134	NQIAIHNDAIAALRK		130.06	2.5	28	AVKLSNMNQRVSRFH		45.81	4
		49	IEVRRVDDDTRVKMI		172.95	4	70	LDRLIQTESRKRQAS		52.32	5
		70	LDRLIQTESRKRQAS		230.44	5	58	TRVKMIKDAIAHLDR		60.7	6
		69	HLDRLIQTESRKRQA		256.55	6	26	ETAVKLSNMNQRVSR		71.81	8
	SALP-1	150	RELRVDNIRGKFEKD	A	71.62	1	189	EIKSLVERHKRSLFE	B	60.69	6
		1	MFSVRADPTKSRLNV	A/B	80.41	1.2	188	DEIKSLVERHKRSLF	B	62.24	6.5
		142	KLNAAIDDRELRVEN	B	240.25	5.5	165	FMRLIESIESSKRAY	A/B	62.53	6.5

		140	AEKLNAAIDDRELRV	A	277.93	6.5	238	ETVMKYLERNIYTGV		64.36	7
		144	LLHQAVKDLDILIQK	A/B	359.35	8.5	52	LDILIQKQTLQRAEA		70.02	7.5
	21.1 protein	355	NQAFKVDLNTLMSTK	A/B	49.95	0.4	142	LNCIKIMLNTHKFTI	B	24.67	1
		341	YEELKQDVAILKAKN	A	57.69	0.6	474	KNEILNEVARRKAAY	A/B	25.08	1.1
			YEELKQDVSILKAKN	B	74.97	1.1	758	EAPIKHSTGRRAIFY	B	27.9	1.4
		551	RKVLTGDKGAMLRHI	A/B	78.95	1.1		EAPIKHSTGRRAIFY	A	27.96	1.4
		683	VRLLLDREAGMTLEN	A	84.7	1.3	142	LNCIKVMLNTHKFTI	A/B	28.49	1.5
		288	NTKLKADLKLQQDQL	B	85.13	1.3	18	HSAIRTAIPRFAGST		28.87	1.5
	α-Tubulin	112	KEIVDLVLDRVRKLA	A/B	28.47	0.06	329	NAAIAVIKTKRTIQF	A/B	13.02	0.09
		153	FLERLSVDYGRKSKL		99.17	1.7	332	IAVIKTKRTIQFVDW		27.03	1.3
		198	SDCAFMVDNEAMYDI		107.64	1.9	394	KFDLMYAKRAFVHWY		31.34	1.8
		238	ITASLRFDGALNVDL		116.79	2.5	112	KEIVDLVLDRVRKLA		35.47	2.5
		110	IGKEIVDLVLDRVRK		126.19	2.5	325	PKDVNAAIAVIKTKR		60.83	6.5
	β-Tubulin	243	PGQLNADLRKLAVNL	A	56.4	0.6	383	EQFTAMFRRKAFLHW	A	28.03	1.4
		61	PRAILVDLEPGTMDS		125.3	2.5	251	RKLAVNLIPFPRLHF		40.87	3.5
		196	ADEVFCIDNEALYDI		225.38	5					
		289	LVSQMFDNKNMMAAS		250.76	5.5	249	DLRKLAVNLIPFPRL		46.3	4
		110	AELVDAVLDVVRKES		321.95	7.5					
	GHSP-115	847	LARLRLRLDESLPAL	A	30.95	0.08	660	EVIKTLRKQLVGKAT	A	17.63	0.4
		153	KAMISHDEKTALILA		48.05	0.4	290	KSAIIQNLERRLQEI		34.28	2.5
		337	YDRILADKDAEISRL		80.34	1.2	424	VARLLAERSALKKRI		38.42	3
		201	SEHVNLDVNMQLIDV		81.35	1.2	235	EARIRQLEMRLVTLH		38.45	3
		1015	YQQLVEDLRAGIATL		96.19	1.6	662	VKMIKDAIAHLDRLI		40.47	3.5
Metabolic Proteins	ADI	88	EREVLMDQAMASLKY	A/B	29.02	0.07	143	EPVIHLIPGVRNTAL	B	21.64	0.7
		495	SREIIADVHKLYQKL	B	29.86	0.07		EPVIHLAPGVRNTAL	A	30.66	1.7
			SREIIADAYGLYQKL	A	122.7	2.5	200	LALIFWKRLGARVVG	A	26.88	1.3
		383	VDFIKADPAYISYCK	A	64.27	0.8		FALIFWKRLGVRVVG	B	32.32	1.9
		506	YQKLISEGRVPYITW	B	145.32	3	123	YKRKVLSALSNRNLV	B	27.37	1.3
		168	NNMVFMRDQQITTRR	A/B	148.87	3		YKRKVLSALSTRNLV	A	30.38	1.7
	OCT	18	KELMYLVDRALDMKK	B	41.1	0.25	45	TLLAFFAKPSLRTRV	A/B	31.58	1.9
			KELAYLIDRALDMKK	A	51.38	0.5	266	DAVMAVTSKRSIFMN	A	32.33	1.9
		259	LTPFQVDDAVMAVTS	A/B	55.52	0.6		DAVMAVTSKRSVFMN	B	32.68	2
		54	SLRTRVSLETAMTRL		183.15	4	44	KTLLAFFAKPSLRTR	A/B	35.44	2.5
		233	GVDVVYTDSWMSYHI		210.67	4.5	47	LAFFAKPSLRTRVSL		36.45	2.5
		299	KSICYDEAGNRLHSA	B	291.37	6.5	268	VMAVTSKRSIFMNCL	A	51.01	5
								VMAVTSKRSVFMNCL	B	53.99	5
	FBA	249	ICKINVDSDSRMAMT	B	31.77	0.09	258	SRMAMTGAIRKVFVE	A	16.51	0.3
			VCKINVDSDSRMAMT	A	32.4	0.1		SRMAMTGAIRKVFAE	B	16.9	0.3
		251	KINVDSDSRMAMTGA	A/B	39.88	0.25	29	EQIQGIMKAVVQLKS	A/B	25.99	1.2
		186	ESDIRLAIDRVKTIS	A	86.77	1.4	33	GIMKAVVQLKSPVIL		32.38	1.9
			ETDIRLAIDRVKTIS	B	87.24	1.4	31	IQGIMKAVVQLKSPV		35.77	2.5
	UPL-1	47	EVKFIRRAPRLFTTI	A/B	81.71	1.3	47	EVKFIRRAPRLFTTI	A/B	10.39	0.02
		114	VGDIIVPDVYRTVLR	A/B	141.22	3	44	PGFEVKFIRRAPRLF		14.83	0.17

		44	PGFEVKFIRRAPRLF		222.31	5	157	LTSIVRKHVAALSYK	B	20.11	0.6
		27	RVLTVGDVPRAVAIA		319.14	7.5		LTSIVRKHVSTLSYK	A	30.35	1.7
							155	HDLTSIVRKHVAALS	B	23.41	0.9
		25	ANRVLTVGDVPRAVA		430.79	10		RDLTSIVRKHVSTLS	A	27.99	1.4
	Enolase	10	IKARMIIDSRGTPTT	A/B	111.43	2	2	EAPSTIKAIKARMII	A/B	9.23	0.01
		425	YNELLRIEDSIKVPY	B	156.21	3.5	68	QALENIRKIIAPALI		22.03	0.7
		251	RMGICIDAAASEFYF	B	191.38	4.5	66	VEQALENIRKIIAPA	A	58.6	6
			LMGICIDAAASEFYS	A	233.98	5.5		VEQALENIRKIITPA	B	67.95	7.5
		2	EAPSTIKAIKARMII	A/B	239.27	5.5	73	IRKIITPALIGMPVR	B	61.35	6.5
VSP	VSPAS8	104	IGLLVCNDRTATADK	A	320.01	7.5	VSP5G8/7	LVAVILQIARAACTP	B	50.4	4.5
	VSP1267	9	ILSTFAVDCKNSGNS		327.05	7.5					
	VSP5	22	SGYFLLDNGCYKTDR	B	359.23	8.5					
	VSP5G8	342	GRALIYGDDPTKGTC		384.35	9	TSA4/7	LAIVILQLARTACTQ	A	47.11	4
	VSP9B10	503	EKCELTVDGTAYCSK	A	396.9	9.5					
HSP	BIP	416/431	HDVLLIDVTPLTLGI	A/B	40.31	0.25	281/296	AKDMAVKKAISRLRR	A/B	16.24	0.25
		102/117	YKVINKDGRPFVQLS		42.34	0.25	280/295	KAKDMAVKKAISRLR		22.05	0.7
		347/362	VEQVLRDAKLKTTDI		70.91	0.9	286/301	VKKAISRLRREIEAG		35.57	2.5
		91/106	PEVQKDMKLLPYKVI		93.65	1.5	133/148	EISAMVLTKMKTIAE		38.91	3
		472/487	GERSMVKDNNLLGNF		129.09	2.5	74/89	ENTIFDVKRLIGRKF		44.68	4
CWPs	CWP 1	60	NNYVIALDLSDMSLT	B	36.35	0.15	109	TNLQYLQINKAGLTG	A	49.3	4.5
			NNYVIALDLSDMGLT	A	42.32	0.25					
		159	LKELHLDCNQLSGTV		102.53	1.7					
	CWP 2	60	NNNVIGIDLSDMGLT	A/B	151.25	3	89	LRTVVRSSSSRASSA	A	29.25	1.6
		125	IPECICDLTHMMFWY		159.01	3.5	290	RTVVRSSSSRASSAT	B	40.39	3.5
	CWP 3	130	LHSLHLDNNSLIGDV	A	178.35	4	154	LGLKLFTARCNRLQY	A	43.65	3.5
Giardia trophozoite antigen	GTA-1	100	LELIMSLAPNHMSAI	A/B	182.16	4	100	LELIMSLAPNHMSAI	A/B	17.37	0.4
		66	DRKALLYEIKRLDRW		237.93	5.5	97	IVTLELIMSLAPNHM		36.88	2.5
		47	SLMLLTEDDLKQMGI		252.39	5.5	103	IMSLAPNHMSAICTV		53.56	5
	GTA-2	109	FVYVASDNRPIPVML		70.58	0.9	24	IRATKVVMVSHLHLR	B	28.07	1.4
		147	SVITDISVDAIQLCI		91.86	1.5	19	TVVNEIRATKVVMVS		28.77	1.5

The epitopes shown are the top 5 binder sequences in MHC class II binding prediction of each protein.

It is considered strong binder > 2% rank and weak binder < 10% rank.
