# Supplementary material for: “Immunoinformatic Identification of T-Cell and B-Cell Epitopes From Giardia lamblia Immunogenic Proteins as Candidates to Develop Peptide-Based Vaccines Against Giardiasis”
Source: Front Cell Infect Microbiol. 2021 Oct 27;11:769446. doi: 10.3389/fcimb.2021.769446 (PMC8579046; doi:10.3389/fcimb.2021.769446)
Supplement: Supplementary file 3 [file DataSheet_3.pdf]

Table S3. Prediction of epitopes from HEL and Ovalbumin proteins for IA^k and IA^d alleles.

		IA^k		
**Protein**	**Position**	**Epitope**	**Affinity (nM)**	**% Rank**
HEL	48	DGSTDYGILQINSRW	1536.81	0.01
	45	RNTDGSTDYGILQIN	7758.19	1.50
	32	AKFESNFNTQATNRN	9465.25	6.00
	100	SDGNGMNAWVAWRNR	9804.97	7.50
	1	KVFGRCELAAAMKRH	9795	7.50
		**IA^d**		
Ovalbumin	319	AESLKISQAVHAAHA	45.16	0.05
	322	LKISQAVHAAHAEIN	66.08	0.20
	29	FYCPIAIMSALAMVY	81.80	0.40
	216	GLFRVASMASEKMKI	110.50	0.80

hen egg-white lysozyme (HEL)
