# Supplementary material for: “Immunoinformatic Identification of T-Cell and B-Cell Epitopes From Giardia lamblia Immunogenic Proteins as Candidates to Develop Peptide-Based Vaccines Against Giardiasis”
Source: Front Cell Infect Microbiol. 2021 Oct 27;11:769446. doi: 10.3389/fcimb.2021.769446 (PMC8579046; doi:10.3389/fcimb.2021.769446)
Supplement: Supplementary file 4 [file DataSheet_4.pdf]

Table S4. Subunit vaccines development of pathogens.

Pathogen	Disease	*In silico* methods	Vaccine candidate	Validation assay		Status	Reference
				In vitro	*In vivo*		
**Protozoa**							
Leishmania infantum	Visceral leishmaniasis	1. MHC-I, MHC-II binding epitope prediction.<br>2. IFN-γ inducing epitopes prediction.<br>3. Subunit vaccine modeling and molecular docking.	Vaccine construct of nine epitopes from four antigenic proteins selected (histone H1, sterol 24-c-methyltrans-ferase, *Leishmania*-specifc hypothetical protein, and *Leishmania*-specifc antigenic protein).	Humoral (IgG2a) and cellular response (Th1 cytokines: IL-2, IFN-γ, TNF-α) activation.	The parasitic load decreases an 88.8% in BALB/c vaccinated.	Pre-clinical	(Vakili et al. 2018, 2020)
		1. Homology of LEISH-F3 construct among *Leishmania* species.	LEISH-F3 Vaccine NH36-SMT antigen (Nucleoside hydrolase NH36 of *L. donovani*, the sterol 24-c-methyltransferase (SMT) from *L. infantum*.	Humoral specific responses (IgG1, IgG2a), CD4+ and CD8+ T cell proliferation by *Leishmania* antigens.	Increases levels of IFN-γ, TNF-α, IL-2, IL-5, and IL-10 indicating Th1 responses in C57BL/6, BALB/c and humans. It also reduces parasite burdens in mice infected.	Clinical Trial Phase I	(Cecílio et al. 2017; Coler et al. 2015)
Trypanosoma cruzi	Chagas	1. MHC-I, MHC-II binding epitope prediction.<br>2. IFN-γ inducing epitopes prediction.<br>3. Linear and conformational B-cell epitope prediction.<br>4. 3-D structure prediction and molecular docking.	Multi-epitope vaccine consisting of 24 epitopes from eight membrane and secretory proteins.	N/E	N/E	Design	(Khatoon et al. 2018)
		1. MHC-I binding epitope prediction.	Pool of ten immunogenic peptides and adjuvant.	Stimulation of splenocytes from BALB/c infected with each individual peptide and IFN-γ secretion.	BALB/c mice immunized shown a lower parasitemia and reduction of cardiac damage.	Pre-clinical	(Teh-Poot et al. 2015)
Plasmodium falciparum	Malaria	1. Homology of circumsporozoite protein among *Plasmoidum falciparum* strains	RTS,S/AS01 vaccine (Mosquirix™). Vaccine comprises conserved sequences of the 3D7 strain of *P. falciparum*.	Neutralizing antibodies production.	Anti- circumsporozoite protein antibody titers and CD4+ T cell responses correlate with a reduced risk of clinical malaria disease in humans.	Clinical Trial Phase III	(Ballou et al. 1987; Dame et al. 1984; Laurens 2020; Neafsey et al. 2015; Zavala et al. 1985)
**Virus**							
Dengue Virus	Dengue	1. The X-ray crystallographic structure of the E-glycoprotein.<br>2. Modelling of interaction with MoAb using the	Tetravalent vaccine V180: Truncated versions (DEN-80E) of the E protein from DENV1-4.	Presence of neutralizing antibodies	Induction of Th1 response, and protective immunity against lethal challenge in mice and non- human primates. In humans induce	Clinical Trial Phase I	(Govindarajan et al. 2015; Manoff et al. 2019)

		defined X-ray diffraction crystal prediction for the tick-borne encephalitis virus E glycoprotein.			neutralizing antibodies, with 85.7% seroconversion		
Influenza virus	Influenza	1. Lists the influenza virus gene products and peptide sequences binding to MHC molecules.	Two candidate vaccines. 1. Nucleocapside-based vaccine with conserved human T cell epitopes for other influenza virus proteins: matrix, non-structural 1, basic polymerase 1, and acidic polymerase. 2. Conserved sequences of neuraminidase and hemagglutinin from influenza viruses.	CD4+ and CD8+ cells stimulation from BALB/c immunized. Cytokines secretion evaluations, IFN-γ, IL2 and TNF-α.	Vaccination delayed mortality by 1–2 days and decreased lung viral titers in mice.	Pre-clinical	(Goodman et al. 2011)
Zika Virus	Zika	1. Homology and phylogenetic analysis among ZIKA variants.	Subunit vaccine: composed of 90% of the N-terminal whole envelope protein.	Increase IgG and neutralizing antibody titers.	In a BALB/c model: Neonatal mice: induces full protection Pregnant mice: fetus and offspring protection from ZIKA-induced microcephaly.	Pre-clinical	(Han et al. 2017; Ye et al. 2016)
			Subunit vaccine: composed of 80% of the N-terminal whole envelope protein.	Increase IgG and neutralizing antibody titers; increase ZIKA specific T cells; IFN-γ	In BALB/c adult mice increases survival rate	Pre-clinical	(Liang et al. 2018; Ye et al. 2016)
SARS-CoV2	COVID-19	1. Modelling S1 and hACE2 structure and interaction.	NovaVax composed of trimeric full-length SARS-CoV-2 spike glycoproteins.	Antibodies produced in animal models blocked hACE2 receptor binding and achieved neutralization of wild-type virus that exceeded the magnitude of responses measured in human convalescent serum	BALB/c and Baboons induced high titers of antibodies anti-spike protein and that provided protection against SARS-CoV-2 challenge. Polyfunctional CD4+ and CD8+ T-cell responses were induced with a T helper 1 (Th1) dominant phenotype	Clinical Trial Phase II	(Keech et al. 2020; Mandolesi et al. 2020; Tian et al. 2021)
**Bacteria**							
Pseudomonas aeruginosa	Otitis, endophthalmitis, endocarditis, meningitis, pneumonia, and septicemia	1. Protein features: No-human- homologus, localization, protein transport, transmembranal sites, function, interactions.	Multi-epitope vaccine consisting of 35 epitopes from 15 outer membrane proteins.	N/E	N/E	Design	(Solanki et al. 2019)

		2. MHC-I, MHC-II binding epitope prediction.<br>3. Linear B-cell epitope prediction.<br>4. Hydropathy of epitopes.<br>5. Constructs evaluation: allergenicity, antigenicity, solubility, physicochemical behavior, prediction of secondary structure, molecular docking and simulation.					
Neisseria meningitidis serogroup B	Meningitis	1. Selection of antigens by features: localization, function, virulence, no-human- homologus.<br>2. Conservation over *Neisseria sp.*<br>3. 3-D structure modeling.	4CMenB Vaccine Multivalent vaccine of four antigens from the epidemic New Zealand strain.	Immunization induced bactericidal antibodies against meningococcal strains representative of the global population diversity	Serum from immunized CD1 mice induced passive protection in vivo of infant Wistar rats from Men B bacteremia	Licensed to use in humans of all ages	(Giuliani et al. 2006; Masignani, Pizza, and Moxon 2019; Rappuoli et al. 2018)
Helicobacter Pylori	Gastric malignancies and peptic ulcers	N/E	Multivalent vaccine with four antigens.	Increased serum antigen-specific IgG responses and mucosal IgA responses.	Oral immunization of *H. pylori* infected mice significantly reduced gastric bacterial colonization at both 2 and 8 weeks after immunization. Induction of Th1/Th17 responses.	Pre-clinical	(Liu et al. 2020)
		1. Protein features: virulence factors<br>2. MHC-I, MHC-II binding epitope prediction.<br>3. Linear and conformational B-cell epitope prediction.<br>4. Constructs evaluation: allergenicity, antigenicity, solubility, physicochemical	Multiepitope vaccine contains 12 epitopes MHC-II and 11 epitopes MHC-I from 4 antigens.	N/E	N/E	Design	(Khan et al. 2019)

		properties, prediction of secondary and tertiary structure.<br>5. Molecular docking and simulation TLR interaction.<br>6. Cloning simulation.					
Mycobacterium Tuberculosis	Tuberculosis	1. T-cell epitopes prediction.	DNA- based multi-epitope vaccine containing five T-cell epitopes casted in 65-kDa heat shock protein.	Humoral immune response (IgG2a/IgG1 ratio). CD4+ and CD8+ cells IFN-gamma, TNF-alpha, IL-2 stimulation.	DNA- based multi-epitope vaccine shown protection in C57BL/6 mice. as well as, decreased lung and spleen viral titers.	Pre-clinical	(Gao et al. 2009)

Abbreviations. Not examinate (N/E), angiotensin-converting enzyme (ACE).

References

Ballou, W. R., S. L. Hoffman, J. A. Sherwood, M. R. Hollingdale, F. A. Neva, W. T. Hockmeyer, D. M. Gordon, I. Schneider, R. A. Wirtz, and J. F. Young. 1987. "Safety and Efficacy of a Recombinant DNA Plasmodium Falciparum Sporozoite Vaccine." *Lancet (London, England)* 1(8545):1277–81.

Cecílio, Pedro, Begoña Pérez-Cabezas, Laura Fernández, Javier Moreno, Eugenia Carrillo, José M. Requena, Epifanio Fichera, Steven G. Reed, Rhea N. Coler, Shaden Kamhawi, Fabiano Oliveira, Jesus G. Valenzuela, Luigi Gradoni, Reinhard Glueck, Gaurav Gupta, and Anabela Cordeiro-da-Silva. 2017. "Pre-Clinical Antigenicity Studies of an Innovative Multivalent Vaccine for Human Visceral Leishmaniasis." *PLoS Neglected Tropical Diseases* 11(11):e0005951.

Coler, Rhea N., Malcolm S. Duthie, Kimberly A. Hofmeyer, Jeffery Guderian, Lakshmi Jayashankar, Julie Vergara, Tom Rolf, Ayesha Misquith, John D. Laurance, Vanitha S. Raman, H. Remy Bailor, Natasha Dubois Cauwelaert, Steven J. Reed, Aarthy Vallur, Michelle Favila, Mark T. Orr, Jill Ashman, Prakash Ghosh, Dinesh Mondal, and Steven G. Reed. 2015. "From Mouse to Man: Safety, Immunogenicity and Efficacy of a Candidate Leishmaniasis Vaccine LEISH-F3+GLA-SE." *Clinical & Translational Immunology* 4(4):e35.

Dame, John B., Jackie L. Williams, Thomas F. McCutchan, James L. Weber, Robert A. Wirtz, Wayne T. Hockmeyer, W. Lee Maloy, J. David Haynes, Imogene Schneider, Donald Roberts, Greg S. Sanders, E. Premkumar Reddy, Carter L. Diggs, and Louis H. Miller. 1984. "Structure of the Gene Encoding the Immunodominant Surface Antigen on the Sporozoite of the Human Malaria Parasite Plasmodium Falciparum." *Science* 225(4662):593–99.

Gao, Haifeng, Kang Li, Shanshan Yu, and Sidong Xiong. 2009. "A Novel DNA Vaccine Containing Multiple TB-Specific Epitopes Cast in a Natural Structure Elicits Enhanced Th1 Immunity Compared with BCG." *Microbiology and Immunology* 53(10):541–49.

Giuliani, Marzia M., Jeannette Adu-Bobie, Maurizio Comanducci, Beatrice Aricò, Silvana Savino, Laura Santini, Brunella Brunelli, Stefania Bambini, Alessia Biolchi, Barbara Capecchi, Elena Cartocci, Laura Ciucchi, Federica Di Marcello, Francesca Ferlicca, Barbara Galli, Enrico Luzzi, Vega Masignani, Davide Serruto, Daniele Veggi, Mario Contorni, Maurizio Morandi, Alessandro Bartalesi, Vanda Cinotti, Donatella Mannucci, Francesca Titta, Elisa Ovidi, Jo Anne Welsch, Dan Granoff, Rino Rappuoli, and Mariagrazia Pizza. 2006. “A Universal Vaccine for Serogroup B Meningococcus.” *Proceedings of the National Academy of Sciences of the United States of America* 103(29):10834–39.

Goodman, Alan G., Paul P. Heinen, Susana Guerra, Aneesh Vijayan, Carlos Oscar S. Sorzano, Carmen E. Gomez, and Mariano Esteban. 2011. “A Human Multi-Epitope Recombinant Vaccinia Virus as a Universal T Cell Vaccine Candidate against Influenza Virus.” *PLOS ONE* 6(10):e25938.

Govindarajan, Dhanasekaran, Steven Meschino, Liming Guan, David E. Clements, Jan H. ter Meulen, Danilo R. Casimiro, Beth-Ann G. Coller, and Andrew J. Bett. 2015. “Preclinical Development of a Dengue Tetravalent Recombinant Subunit Vaccine: Immunogenicity and Protective Efficacy in Nonhuman Primates.” *Vaccine* 33(33):4105–16.

Han, Jian-Feng, Yang Qiu, Jiu-Yang Yu, Hong-Jiang Wang, Yong-Qiang Deng, Xiao-Feng Li, Hui Zhao, Han-Xiao Sun, and Cheng-Feng Qin. 2017. “Immunization with Truncated Envelope Protein of Zika Virus Induces Protective Immune Response in Mice.” *Scientific Reports* 7(1):10047.

Keech, Cheryl, Gary Albert, Iksung Cho, Andreana Robertson, Patricia Reed, Susan Neal, Joyce S. Plested, Mingzhu Zhu, Shane Cloney-Clark, Haixia Zhou, Gale Smith, Nita Patel, Matthew B. Frieman, Robert E. Haupt, James Logue, Marisa McGrath, Stuart Weston, Pedro A. Piedra, Chinar Desai, Kathleen Callahan, Maggie Lewis, Patricia Price-Abbott, Neil Formica, Vivek Shinde, Louis Fries, Jason D. Lickliter, Paul Griffin, Bethanie Wilkinson, and Gregory M. Glenn. 2020. “Phase 1–2 Trial of a SARS-CoV-2 Recombinant Spike Protein Nanoparticle Vaccine.” *New England Journal of Medicine* 383(24):2320–32.

Khan, Mazhar, Shahzeb Khan, Asim Ali, Hameed Akbar, Abrar Mohammad Sayaf, Abbas Khan, and Dong-Qing Wei. 2019. “Immunoinformatics Approaches to Explore Helicobacter Pylori Proteome (Virulence Factors) to Design B and T Cell Multi-Epitope Subunit Vaccine.” *Scientific Reports* 9(1):13321.

Khatoon, Nazia, Rupal Ojha, Amit Mishra, and Vijay Kumar Prajapati. 2018. “Examination of Antigenic Proteins of Trypanosoma Cruzi to Fabricate an Epitope-Based Subunit Vaccine by Exploiting Epitope Mapping Mechanism.” *Vaccine* 36(42):6290–6300.

Laurens, Matthew B. 2020. “RTS,S/AS01 Vaccine (Mosquirix™): An Overview.” *Human Vaccines & Immunotherapeutics* 16(3):480–89.

Liang, Huabin, Ruoheng Yang, Zhihua Liu, Min Li, Haitao Liu, and Xia Jin. 2018. “Recombinant Zika Virus Envelope Protein Elicited Protective Immunity against Zika Virus in Immunocompetent Mice.” *PloS One* 13(3):e0194860.

Liu, Meiying, Youxiu Zhong, Jing Chen, Yu Liu, Chongfa Tang, Xuewei Wang, Yanbin Zhang, Ping Wang, Susan M. Logan, Wangxue Chen, and Bo Wei. 2020. “Oral Immunization of Mice with a Multivalent Therapeutic Subunit Vaccine Protects against Helicobacter Pylori Infection.” *Vaccine* 38(14):3031–41.

Mandolesi, Marco, Daniel J. Sheward, Leo Hanke, Junjie Ma, Pradeepa Pushparaj, Laura Perez Vidakovics, Changil Kim, Karin Loré, Xaquin Castro Dopico, Jonathan M. Coquet, Gerald McInerney, Gunilla B. Karlsson Hedestam, and Ben Murrell. 2020. "SARS-CoV-2 Protein Subunit Vaccination Elicits Potent Neutralizing Antibody Responses." *BioRxiv* 2020.07.31.228486.

Manoff, Susan B., Michele Sausser, Amy Falk Russell, Jason Martin, David Radley, Donna Hyatt, Christine C. Roberts, Jason Lickliter, Janakan Krishnarajah, Andrew Bett, Sheri Dubey, Tyler Finn, and Beth-Ann Coller. 2019. "Immunogenicity and Safety of an Investigational Tetravalent Recombinant Subunit Vaccine for Dengue: Results of a Phase I Randomized Clinical Trial in Flavivirus-Naïve Adults." *Human Vaccines & Immunotherapeutics* 15(9):2195–2204.

Masignani, Vega, Mariagrazia Pizza, and E. Richard Moxon. 2019. "The Development of a Vaccine Against Meningococcus B Using Reverse Vaccinology." *Frontiers in Immunology* 10:751.

Neafsey, Daniel E., Michal Juraska, Trevor Bedford, David Benkeser, Clarissa Valim, Allison Griggs, Marc Lievens, Salim Abdulla, Samuel Adjei, Tsiri Agbenyega, Selidji T. Agnandji, Pedro Aide, Scott Anderson, Daniel Ansong, John J. Aponte, Kwaku Poku Asante, Philip Bejon, Ashley J. Birkett, Myriam Bruls, Kristen M. Connolly, Umberto D'Alessandro, Carlota Dobaño, Samwel Gesase, Brian Greenwood, Jonna Grimsby, Halidou Tinto, Mary J. Hamel, Irving Hoffman, Portia Kamthunzi, Simon Kariuki, Peter G. Kremsner, Amanda Leach, Bertrand Lell, Niall J. Lennon, John Lusingu, Kevin Marsh, Francis Martinson, Jackson T. Molel, Eli L. Moss, Patricia Njuguna, Christian F. Ockenhouse, Bernhards Ragama Ogutu, Walter Otieno, Lucas Otieno, Kephas Otieno, Seth Owusu-Agyei, Daniel J. Park, Karell Pellé, Dana Robbins, Carsten Russ, Elizabeth M. Ryan, Jahit Sacarlal, Brian Sogoloff, Hermann Sorgho, Marcel Tanner, Thor Theander, Innocent Valea, Sarah K. Volkman, Qing Yu, Didier Lapierre, Bruce W. Birren, Peter B. Gilbert, and Dyann F. Wirth. 2015. "Genetic Diversity and Protective Efficacy of the RTS,S/AS01 Malaria Vaccine." *New England Journal of Medicine* 373(21):2025–37.

Rappuoli, Rino, Mariagrazia Pizza, Vega Masignani, and Kumaran Vadivelu. 2018. "Meningococcal B Vaccine (4CMenB): The Journey from Research to Real World Experience." *Expert Review of Vaccines* 17(12):1111–21.

Solanki, Vandana, Monalisa Tiwari, and Vishvanath Tiwari. 2019. "Prioritization of Potential Vaccine Targets Using Comparative Proteomics and Designing of the Chimeric Multi-Epitope Vaccine against Pseudomonas Aeruginosa." *Scientific Reports* 9(1):5240.

Teh-Poot, Christian, Evelyn Tzec-Arjona, Pedro Martínez-Vega, Maria Jesus Ramirez-Sierra, Miguel Rosado-Vallado, and Eric Dumonteil. 2015. "From Genome Screening to Creation of Vaccine against Trypanosoma Cruzi by Use of Immunoinformatics." *Journal of Infectious Diseases* 211(2):258–66.

Tian, Jing-Hui, Nita Patel, Robert Haupt, Haixia Zhou, Stuart Weston, Holly Hammond, James Logue, Alyse D. Portnoff, James Norton, Mimi Guebre-Xabier, Bin Zhou, Kelsey Jacobson, Sonia Maciejewski, Rafia Khatoon, Malgorzata Wisniewska, Will Moffitt, Stefanie Kluepfel-Stahl, Betty Ekechukwu, James Papin, Sarathi Boddapati, C. Jason Wong, Pedro A. Piedra, Matthew B. Frieman, Michael J. Massare, Louis Fries, Karin Lövgren Bengtsson, Linda Stertman, Larry Ellingsworth, Gregory Glenn, and Gale Smith. 2021. "SARS-CoV-2 Spike Glycoprotein Vaccine Candidate NVX-CoV2373 Immunogenicity in Baboons and Protection in Mice." *Nature Communications* 12(1):372.

Vakili, Bahareh, Mahboobeh Eslami, Gholam Reza Hatam, Bijan Zare, Nasrollah Erfani, Navid Nezafat, and Younes Ghasemi. 2018. "Immunoinformatics-Aided Design of a Potential Multi-Epitope Peptide Vaccine against Leishmania Infantum." *International Journal of Biological Macromolecules* 120(Pt A):1127–39.

Vakili, Bahareh, Navid Nezafat, Bijan Zare, Nasrollah Erfani, Maryam Akbari, Younes Ghasemi, Mohammad Reza Rahbar, and Gholam Reza Hatam. 2020. "A New Multi-Epitope Peptide Vaccine Induces Immune Responses and Protection against Leishmania Infantum in BALB/c Mice." *Medical Microbiology and Immunology* 209(1):69–79.

Ye, Qing, Zhong-Yu Liu, Jian-Feng Han, Tao Jiang, Xiao-Feng Li, and Cheng-Feng Qin. 2016. "Genomic Characterization and Phylogenetic Analysis of Zika Virus Circulating in the Americas." *Infection, Genetics and Evolution* 43:43–49.

Zavala, F., J. P. Tam, M. R. Hollingdale, A. H. Cochrane, I. Quakyi, R. S. Nussenzweig, and V. Nussenzweig. 1985. "Rationale for Development of a Synthetic Vaccine against Plasmodium Falciparum Malaria." *Science (New York, N.Y.)* 228(4706):1436–40.
